# Benefits of near-universal vaccination and treatment access to manage COVID-19 burden in the United States

**DOI:** 10.1186/s12916-023-03025-z

**Published:** 2023-08-24

**Authors:** Fuhan Yang, Thu Nguyen-Anh Tran, Emily Howerton, Maciej F. Boni, Joseph L. Servadio

**Affiliations:** Department of Biology and Center for Infectious Disease Dynamics, Pennsylvania State University, University Park, PA 16802 USA

**Keywords:** SARS-CoV-2, COVID-19, Mortality, Vaccination, Treatment, Epidemic modeling

## Abstract

**Background:**

As we continue the fourth year of the COVID-19 epidemic, SARS-CoV-2 infections still cause high morbidity and mortality in the United States. During 2020–2022, COVID-19 was one of the leading causes of death in the United States and by far the leading cause among infectious diseases. Vaccination uptake remains low despite this being an effective burden reducing intervention. The development of COVID-19 therapeutics provides hope for mitigating severe clinical outcomes. This modeling study examines combined strategies of vaccination and treatment to reduce the burden of COVID-19 epidemics over the next decade.

**Methods:**

We use a validated mathematical model to evaluate the reduction of incident cases, hospitalized cases, and deaths in the United States through 2033 under various levels of vaccination and treatment coverage. We assume that future seasonal transmission patterns for COVID-19 will be similar to those of influenza virus and account for the waning of infection-induced immunity and vaccine-induced immunity in a future with stable COVID-19 dynamics. Due to uncertainty in the duration of immunity following vaccination or infection, we consider three exponentially distributed waning rates, with means of 365 days (1 year), 548 days (1.5 years), and 730 days (2 years). We also consider treatment failure, including rebound frequency, as a possible treatment outcome.

**Results:**

As expected, universal vaccination is projected to eliminate transmission and mortality. Under current treatment coverage (13.7%) and vaccination coverage (49%), averages of 81,000–164,600 annual reported deaths, depending on duration of immunity, are expected by the end of this decade. Annual mortality in the United States can be reduced below 50,000 per year with 52–80% annual vaccination coverage and below 10,000 annual deaths with 59–83% annual vaccination coverage, depending on duration of immunity. Universal treatment reduces hospitalizations by 88.6% and deaths by 93.1% under current vaccination coverage. A reduction in vaccination coverage requires a comparatively larger increase in treatment coverage in order for hospitalization and mortality levels to remain unchanged.

**Conclusions:**

Adopting universal vaccination and universal treatment goals in the United States will likely lead to a COVID-19 mortality burden below 50,000 deaths per year, a burden comparable to that of influenza virus.

**Supplementary Information:**

The online version contains supplementary material available at 10.1186/s12916-023-03025-z.

## Background

The COVID-19 epidemic in the United States entered its fourth year on March 1, 2023, with 507,000 Americans dying in the first year, 433,000 in the second year, and 136,000 in the third year [1]. The epidemic response focused initially on non-pharmaceutical interventions (NPIs), treatment of severe cases, vaccine rollout in early 2021, and widespread rapid testing by late 2021 [2]. However, none of these interventions have allowed the US COVID-19 death rate to fall below 100 deaths per day even during non-peak periods of transmission. This would be required to lower COVID deaths to an annual number near 50,000 (< 20/100,000 annual mortality incidence), comparable to a severe influenza season [3]. Such a goal is reasonable for the US, a wealthy country with advanced public and private sectors in medicine and public health. To achieve a substantial mortality reduction in COVID-19 in the next several years, the most probable path lies in our past successes at controlling vaccine-preventable diseases: actively promoting universal or near-universal vaccine coverage rather than issuing recommendations for voluntary individual vaccine uptake.

The unique challenge in long-term planning of SARS-CoV-2 vaccination is that it conforms neither to our past experiences eliminating childhood vaccine-preventable infections such as smallpox, polio, or measles, nor can it be modeled on our current strategy to promote voluntary influenza vaccination which is meant to reduce risk of hospitalization and death in the youngest and oldest age groups. The clinical burden of SARS-CoV-2 is concentrated in the oldest age groups where population mortality rates are several times higher than those for influenza [4, 5]. Thus, it is likely that past approaches of universal childhood vaccination or voluntary vaccination, i.e., without urgent recommendations and proactive planning, will not work at substantially reducing the annual COVID-19 mortality burden.

Currently, there is substantial evidence that available COVID-19 vaccines are safe and effective at reducing transmission and decreasing the severity of disease [6, 7]. Modeling studies have shown that increasing vaccination coverage is beneficial for population health [8, 9], with some evidence in favor of targeting vulnerable populations in particular [10] and of combining vaccination with NPIs [11]. Similarly, current treatments have been shown to reduce the severity of disease, the likelihood of hospitalization, and the duration of infection [12–15]. This is in contrast to other respiratory viruses, where treatments are not widely available [16]. Therefore—with appropriate supply, access, cost, and coverage—it may be possible to substantially reduce annual SARS-CoV-2 mortality rates in the US through therapeutic interventions in the current context of voluntary vaccination. However, fewer studies have aimed to examine how to use both vaccination and therapeutics to successfully reduce mortality. It is not known what combination of increased vaccination rates and increased access to anti-COVID therapeutics would be the most cost-effective, and it is likewise not known what level of coverage of each is required to reduce annual death counts to levels similar to or lower than those for influenza virus.

This study aims to evaluate the effectiveness of different strategies using antiviral medications in tandem with higher levels of vaccination to adequately reduce COVID-19 morbidity and mortality in future epidemic years. We adapted a previously validated mathematical model of SARS-CoV-2 epidemiology and clinical progression in the US to predict future burden and then implemented various strategies of vaccine and antiviral deployments. We compared effectiveness of strategies via reductions in case totals, hospitalizations, and deaths. The results of this study provide insight into the benefits of using both annual vaccines and antiviral medications to reduce long-term COVID-19 burden.

## Methods

This study uses a previously published dynamical model (Additional file 1: Fig. S1) [17–19] designed to examine COVID-19 burden in Rhode Island (RI), Massachusetts, Connecticut, and Pennsylvania. The model incorporates key aspects of SARS-CoV-2 dynamics, including asymptomatic transmission, vaccination, and age-specific (10-year age bands) risks of infection and severe outcomes. The model also accounts for underreporting of disease burden through a reporting rate, allowing the infected classes to represent reported symptomatic cases. Eleven daily data streams were used for the Bayesian model fit, which showed consistency in its inference of clinical and epidemiological transition parameters across four different states and at different phases of data collection [17, 18]. We adapted the previously published model, and based on its previous fit for RI [17, 18], we calibrated the transmission rate for the US and produced simulations for the present study.

### Data sources

Daily data for cumulative cases, hospitalizations, and deaths are publicly available from the RI Department of Health (DOH) [20]. From these, we calculated daily incident cases, hospital admissions, and deaths. To apply results from a model calibrated to RI to the entire US, we collected weekly incident cases and deaths for the US, which are publicly available from the US Centers for Disease Control and Prevention (CDC) [1], and weekly hospitalization data, which are publicly available from the Department of Health and Human Services (HHS) [21]. These data are available by state and were summed to represent national burden. For both RI and the US, we collected data between March 1, 2020, and November 30, 2022.

COVID-19 vaccination data for RI are publicly available from RI DOH and CDC. Weekly counts of people who completed the doses of a primary series (two doses of Pfizer or Moderna vaccines or single dose of Johnson and Johnson vaccine) from RI are available from RI DOH [20] from December 13, 2020, to July 30, 2022. The data afterwards are available from CDC [22] through November 30, 2022. Both data sources contain age-specific totals of individuals receiving a COVID-19 vaccine (either an initial series or booster). The age groups from CDC were as follows: 0–4, 5–12, 13–17, 18–64, 65 + , and these were adjusted to our 10-year age bands assuming a uniform distribution of coverage within each CDC age band.

Monthly influenza vaccination coverage from the 2010–2011 season to the 2020–2021 season was collected from the CDC [23], provided by the National Immunization Survey-Flu (NIS-Flu) and the Behavioral Risk Factor Surveillance System (BRFSS). Coverage was estimated as the proportion of the US population that received an influenza vaccine based on telephone surveys conducted from October to May in each season. Coverage among children (6 months–17 years old) is reported by NIS-Flu, and coverage among adults is reported by BRFSS.

The national and jurisdictional cumulative counts of delivered and administered therapeutics including nirmatrelvir/ritonavir (Paxlovid), molnupiravir (Lagevrio), and tixagevimab/cilgavimab (Evusheld) are available from HHS [24]. We defined treatment coverage in the current season as the coverage of Paxlovid because it is the most commonly used COVID-19 therapeutic in the US. Current treatment coverage is 13.7%, calculated from total administered doses of Paxlovid (6,279,116) and total COVID-19 cases (45,666,906) between January 1, 2022, and November 30, 2022, in the US.

### Model adaptation

We introduced one vaccination class to the previously developed model. The vaccine provides protection against infection: after receiving a vaccine, individuals are moved to this class (“Vac” in Additional file 1: Fig. S1). The vaccine only protects individuals who are not infected, including individuals who recently recovered or were discharged from hospital, and susceptible individuals. While we allow individuals who are infected and not yet symptomatic to get vaccinated, including individuals who are presymptomatic or asymptomatic, they will continue a normal course of infection. We assumed that mRNA vaccines have around 95% efficacy against infection for age > 18 based on previous studies [25, 26]. Thus, we assume 95% vaccine efficacy against infection for all ages. From December 13, 2020, to November 30, 2022, the vaccination rate was determined by the weekly number of people who completed a primary series (two doses from Pfizer or Moderna, or one dose from Johnson & Johnson) reported by RI DOH or CDC.

To account for treatment of symptomatic individuals, additional transitions were added between classes. During the 6-day infection period, symptomatic individuals who receive treatment will do so during the first 3 days of infection. As a rebounding effect (the relapse of symptoms or viral load within a short time after treatment) of the therapeutics has been reported [27–32], we consider three treatment outcomes: (*i*) successful treatment, where patients recover completely after treatment and move to the recovered class after day 3 of infection; (*ii*) treatment failure, which includes rebound, where patients are still symptomatic and infectious after failed treatment; and (*iii*) hospitalization, where patients are hospitalized after failed treatment. Treatment efficacy is modeled by two parameters: probability of treatment failure and hospitalization fraction given treatment failure. We set the probability of treatment failure to be 5.9% based on population studies on the rebounding effect of Paxlovid and Molnupiravir [32, 33] and the probability of hospitalization after failed treatment to an 88% risk reduction of hospitalization when compared to no treatment [12, 34]. Other values for these parameters are discussed in the “Potential risks of vaccine or treatment failure” section.

We introduced waning of infection-induced and vaccine-induced immunity starting at the beginning of the Delta period (June 2021). A number of studies have been published comparing times to reinfection among individuals who have or have not been previously vaccinated or infected [35–42] many of which align with an estimated 40% to 70% becoming susceptible to reinfection after 1 year (Table S1). Comparison among studies and inference on absolute measures of protection are challenging as the studies (*i*) used different controls groups and (*ii*) used cohorts that were exposed to infection during different periods of the epidemic. Nevertheless, the rates of reinfection in these studies appear to be consistent with average rates of immune waning between 365 days (1 year) and 730 days (2 years), so we considered both durations as well as 548 days (1.5 years) in analyses. Exponentially distributed waning periods with mean durations of 365 days, 548 days, and 730 days imply that 63%, 49%, and 39% of individuals lose immunity within 1 year of infection, respectively.

Since we project disease burden for a long duration (8 years), we also incorporated natural births, deaths, and aging in the model. The national natural birth and death rates were based on recent CDC reports [43, 44].

Parameters pertaining to clinical progression, such as probability of symptomatic infection, hospitalization, and death, were previously fit for RI using data through June 6, 2021 [18]; we used these values in the model for this time period. These, as well as parameters for vaccine and treatment efficacies [45], were updated based on published literature for the Delta period (June 7, 2021–December 20, 2021) and Omicron period (December 21, 2021 onward) [46–51]. Details are presented in Table S2.

Between June 7, 2021, and November 30, 2022, we calibrated the time-varying transmission rate to fit the daily hospitalized cases in the US such that at least 85% of the days within this period produced modeled hospitalization values within 10% of the observed hospitalization incidence. Across the three transmission assumptions, the weeks falling outside this window occurred in June, July, and August of 2021 and May, June, and November of 2022. We chose to fit hospitalization data because it is less affected by underreporting. We then applied the average transmission rate parameter in the Omicron period (December, 21, 2021, to November, 30, 2022) to December 1, 2022, through February 28, 2023, multiplied by a factor such that the observed deaths between March 1, 2022, and February 28, 2023, would be approximately 170,000, reflecting the probable number of deaths during this period (162,136 between March 2, 2022, and January 31, 2023, as of February 7, 2023) [1]. To apply results from a RI-calibrated model to the entire US, we scaled model outputs using the ratio of the total reported symptomatic cases between the US and RI during each of the four variant periods: wildtype (March 1, 2020–March 30, 2021), Alpha (March 31, 2021–June 6, 2021), Delta (June 7, 2021–December 20, 2021), Omicron (December 21, 2021 onward). We used this method rather than scaling by population size because RI contributes a substantially higher number of cases compared to population. The US population is 330 times larger than the RI population, and US case numbers were between 220 and 240 times higher than RI case numbers; this is likely due to higher population density and higher-than-average reporting in RI. This method is also equivalent to comparing population-based incidence for RI and the US. The scaled-up results match the trends observed in the US (Additional file 1: Fig. S2).

Using current coverage of vaccination and treatment, we established a “status quo” scenario, representing COVID-19 projections if vaccination and treatment use remain unchanged. We set the status quo treatment coverage at 13.7% and the initial vaccination coverage to 49% based on the allocation of Paxlovid and the administered number of primary series and boosters during the third year of the epidemic (2022–2023) (detailed assumption of vaccine coverage are in Additional file 1: S1 Text, Table S3, and Fig. S3). Current statewide levels of treatment and vaccination are available in Additional file 1: Fig. S4 and Additional file 1: Fig. S5.

### Projecting future disease burden

We assumed future transmission would be equal to the average over the observed Omicron period (December 21, 2021–November 30, 2022) and adjusted the transmission levels in the model for future projections based on influenza dynamics in the US. Seasonal wintertime forcing in transmissibility was introduced starting in the 2023–2024 season, where transmission increases by up to 20% in a sinusoidal curve between October and February, estimated from influenza transmission during winter in the northeastern US [52]. We assumed monthly COVID-19 vaccination patterns would resemble those of influenza vaccinations. Age-specific proportions of influenza vaccines administered during each month were linearly interpolated to generate age-specific proportions of administered COVID-19 vaccines administered each week in a year (Details in Additional file 1: S1 Text). The annual age-specific trend of vaccination is shown in Additional file 1: Fig. S6.

From the status quo, we generated model projections from March 1, 2023, to February 28, 2033, and recorded cumulative reported cases, hospital admissions, and deaths. We then applied changes in vaccine coverage and treatment coverage to the model to estimate burden under different strategies of vaccination and/or treatment. Because the epidemic showed transient dynamics over the first two seasons (2023, 2024) and settled down to stationary behavior starting in 2025, we used averaged annual cases, hospital admissions, and deaths over 2025–2033 as the primary measure for comparison across strategies. We repeated analyses for two additional transmissibility scenarios, optimistic and pessimistic, referring to transmissibility equal to half and double, respectively, of that observed during the 2022–2023 season.

## Results

### Status quo projections

If current vaccination and treatment coverage (49% and 13.7%, respectively) do not change going forward and a neutral assumption regrading transmission (average transmission from 2022–2023 stays the same) holds, our model projects an average annual COVID-19 burden of 12.7 million reported cases, 485,000 hospitalizations, and 81,000 deaths for 730-day immune waning; 21.3 million reported cases, 807,000 hospitalizations, and 127,000 deaths for 548-day immune waning, and an annual burden of 30.1 million reported cases, 1,111,000 hospitalizations, and 164,000 deaths for 365-day immune waning. In an “optimistic” scenario where future transmission rates are half of the estimated transmission rates for the 2022–2023 season, yearly burden ranges from 1.3 million to 7.3 million cases, 47,000 to 224,000 hospitalizations, and 6689 to 29,000 deaths (across the three immune waning rates). In a “pessimistic” scenario where transmission is doubled, yearly burden is estimated to be between 20.8 million and 50.6 million cases, 917,000 and 2.2 million hospitalizations, and 167,000 and 362,000 deaths. The neutral and pessimistic scenarios both predict mortality from COVID-19 that substantially exceeds that from influenza seasons (range of annual mortality: 12,000–52,000 [3]) if no increases are made to vaccination or treatment coverage.

### Increasing vaccination coverage

**Fig. 1:**
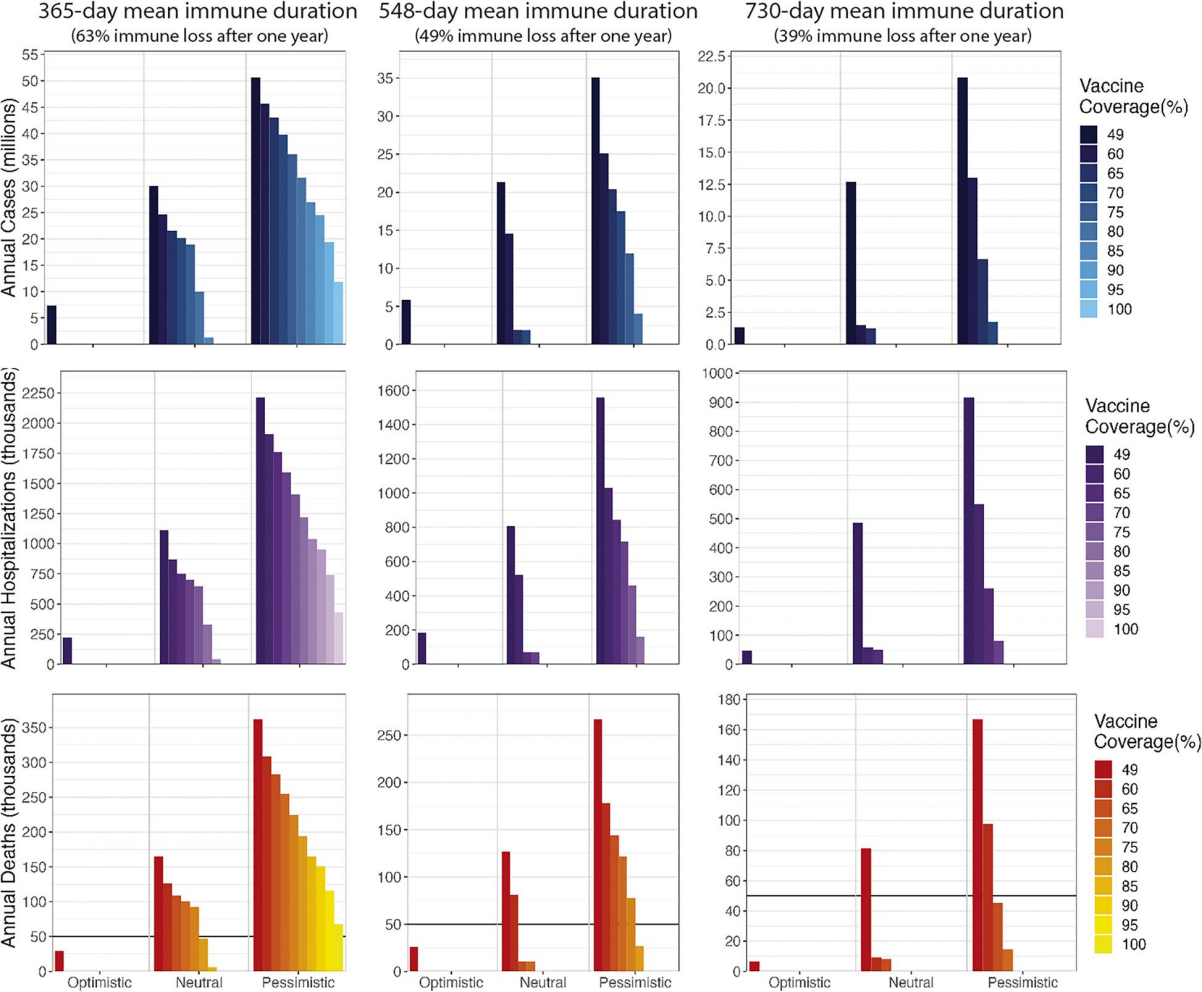
Annual cases, hospitalizations, and deaths between 2025 and 2033 under varying vaccination coverages in three transmission scenarios and three immune durations. The current vaccination coverage is 49%. The treatment coverage through the entire period is 13.7%

Assuming treatment coverage remains at current levels, increasing vaccination coverage alone reduces cases, hospitalizations, and deaths. When the transmission rate stays the same as the 2022–2023 season (neutral scenario), our model shows that annual deaths from COVID-19 during the next 5–10 years can be reduced to fewer than 50,000 (comparable to a severe influenza season) if more than 52% (730-day immune warning), 62% (548-day immune waning), or 80% (365-day immune waning) of the population is vaccinated every year. Deaths can be reduced to fewer than 10,000 if more than 59% (730-day immune waning), 72% (548-day immune waning), or 83% (365-day immune waning) of the population is vaccinated every year (Fig. 1). In these neutral scenarios, disease burden would be eliminated by the start of the 2025–2026 season if annual vaccination reaches 68% (730-day immune warning), 76% (548-day immune waning), or 90% (365-day immune waning) coverage, respectively.

If future transmissibility is halved, current vaccination coverage (49%) would reduce deaths below 50,000 for all three durations of immune waning. Increasing the coverage to 56% (365-day immune duration), 52% (548-day immune duration) would reduce annual deaths below 10,000 by the 2025–2026 season, which is achieved with 49% coverage if the immune duration is 730 days. Increasing coverage to 58% (730-day immune duration), 61% (548-day immune duration), and 68% (365-day immune duration) would eliminate mortality at the beginning of the 2025–2026 season. In the pessimistic scenario where transmissibility is doubled, even universal vaccination coverage is not projected to reduce annual mortality below 50,000 if the true mean duration of immune waning is 365 days. Annual vaccination coverage needs to reach 78% for 548-day immune duration and 65% for 730-day immune duration to achieve fewer than 50,000 deaths per year. Eighty-two percent for 548-day immune duration or 72% for 730-day immune duration is needed to reduce annual deaths below 10,000. To eliminate mortality, 89% (548-day immune duration) or 78% (730-day immune duration) annual vaccine coverage is needed to eliminate mortality as we enter the 2025–2026 season.

### Increasing treatment coverage

**Fig. 2:**
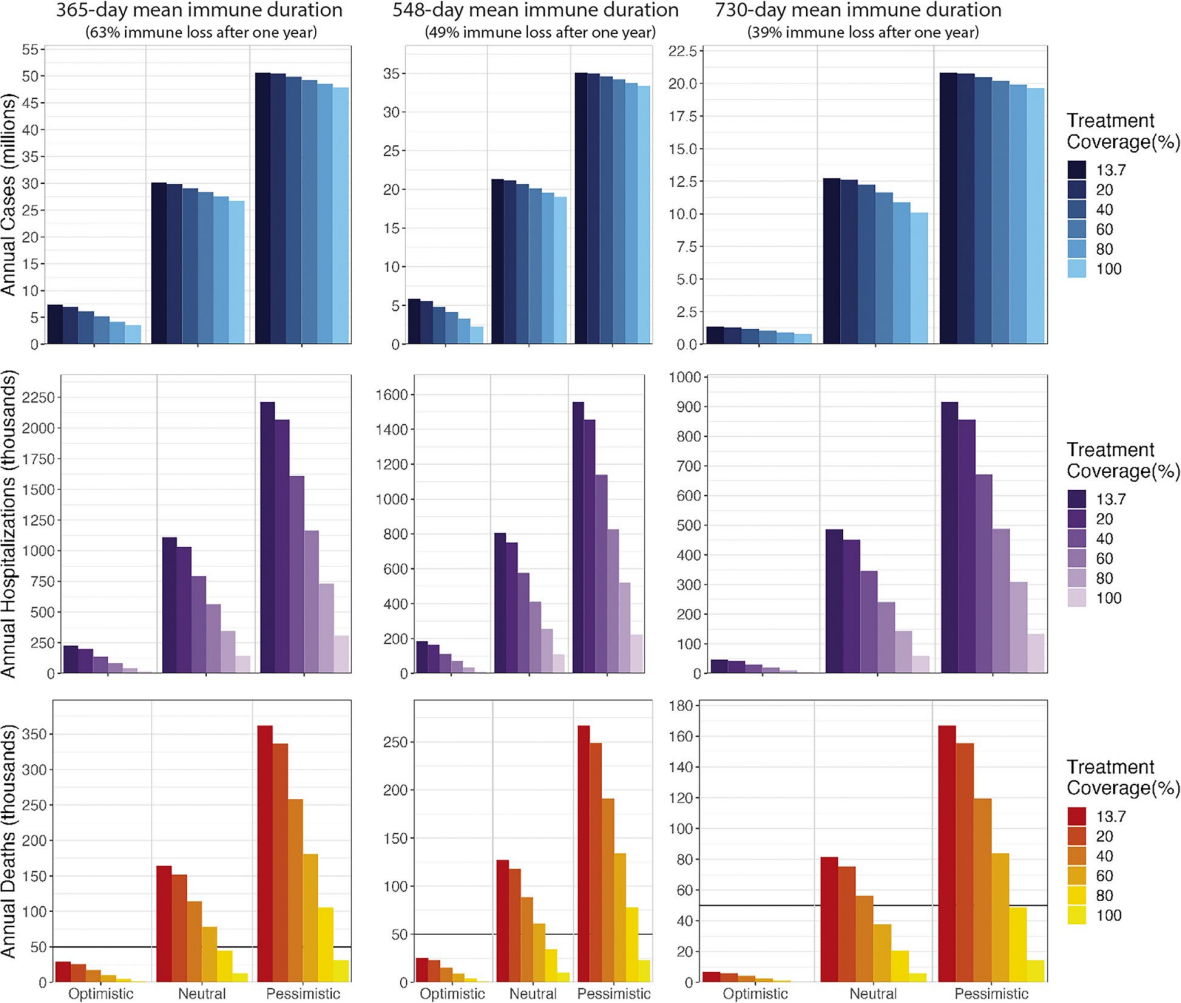
Annual cases, hospitalizations, and deaths between 2025 and 2033 under varying treatment coverage in three transmission scenarios and three immune durations. The current treatment coverage is 13.7%. The vaccination coverage through the entire period is 49%

Assuming current vaccination coverage and virus transmissibility remain unchanged, treatment coverage or treatment access rates of 47% (730-day immune duration), 69% (548-day immune duration), or 77% (365-day immune duration) would be sufficient to reduce annual death rates below 50,000. One hundred percent treatment coverage cannot reduce the annual death below 10,000 under 365-day and 548-day. Ninety-five percent treatment coverage would reduce the annual death below 10,000 under 730-day. Benefits of universal treatment are consistent across the three transmission scenarios and three immune durations with average reductions of 88.6% for hospitalizations and 93.1% for deaths. Increasing population-level treatment coverage reduces disease burden in a linear manner (Fig. 2) and is more effective at reducing hospitalizations and deaths than infections. We project that a 10% increase (absolute percentage points) in treatment coverage leads to an average annual reduction of 390,000 cases, 111,000 hospitalizations, and 17,400 deaths (365-day immune waning); a reduction of 265,000 cases, 80,000 hospitalizations, and 13,400 deaths (548-day immune duration); or a reduction of 312,000 cases, 49,000 hospitalizations, and 8,700 deaths (730-day immune waning). As expected, access to treatment becomes more important as vaccination coverage declines (Additional file 1: Fig. S7) and if future transmission rates increase (Fig. 2); though the relative decrease is similar across scenarios, the absolute difference changes substantially.

### Combining strategies of vaccination and treatment coverage

**Fig. 3:**
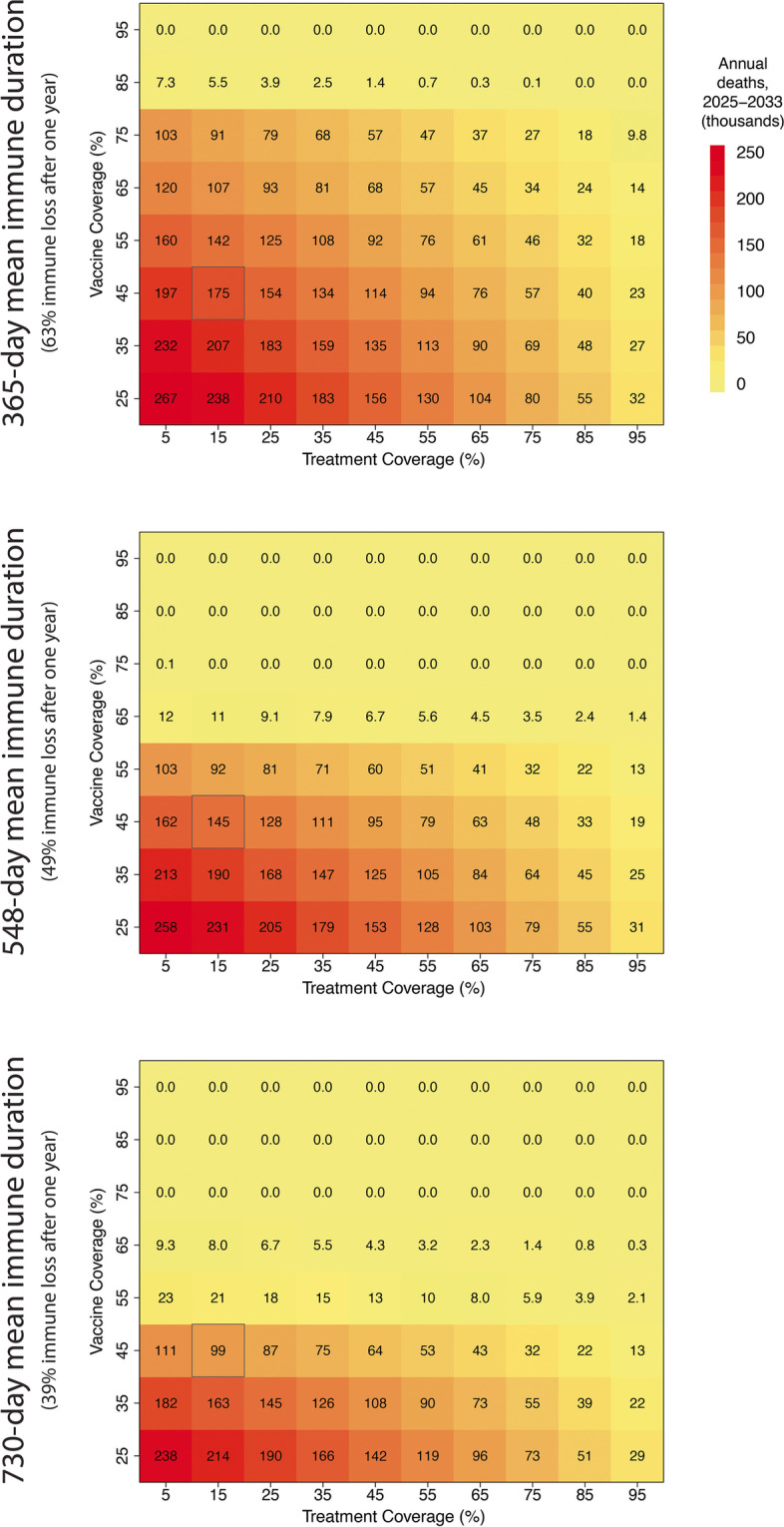
Mean annual deaths (in thousands) between 2025 and 2033 under various combinations of vaccination and treatment coverage. Three rates of immune waning are considered: 365 days (top), 548 days (middle), and 730 days (bottom). The outlined cell represents the treatment and vaccination coverages closest to observed levels (49% vaccine coverage, 13.7% treatment coverage)

We varied treatment and vaccine coverage simultaneously to identify combinations that lower annual death counts to more acceptable levels. For example, if vaccination coverage can only be increased to 55%, then 55% vaccine coverage with 65–75% treatment coverage (depending on immune durations) would reduce annual death counts below 50,000 (Fig. 3, Additional file 1: Fig. S8). The accrual of public health benefits is linear with increasing treatment coverage but non-linear (accelerating) with increasing vaccination coverage. Sufficiently high vaccination rates will eliminate deaths altogether regardless of treatment coverage, but the same is not true for sufficiently high treatment access. Multiple combinations of vaccine/treatment access are projected to lead to annual death counts below 50,000; as examples, for 548-day immune duration, 55%/65% access, 45%/75% access, and 35%/85% access are all associated with ~ 50,000 annual deaths (Fig. 3). A small drop in vaccination coverage requires a comparatively larger compensatory increase in treatment coverage to achieve the same mortality result. Similar effects of vaccination and treatment are observed in reducing hospitalization (Additional file 1: Fig. S9) or cases (Additional file 1: Fig. S10).

**Fig. 4:**
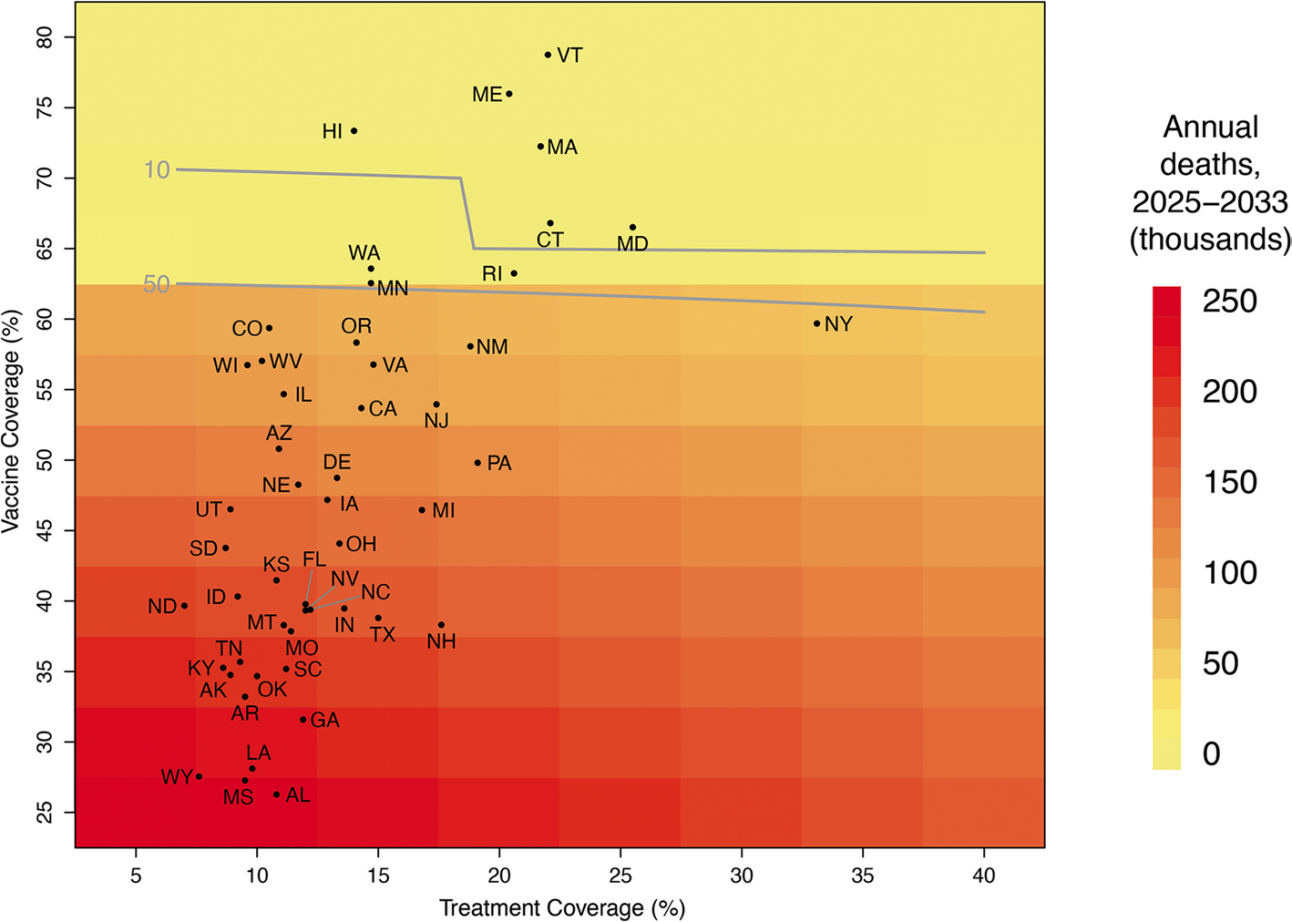
Estimated annual burden between 2025 and 2033 under 548-day immunity waning if implementing the current vaccination and treatment coverages of 50 states nationally (black dots). Contour lines show 10,000 and 50,000 annual deaths, representing the highest and lowest death counts from recently observed influenza seasons

Current vaccine and treatment coverages by state serve as examples of achievable coverage levels at the national level (Additional file 1: Fig. S4, Additional file 1: Fig. S5). Additionally, this information identifies states requiring the most effort and highest resource allocation to improve coverage rates, as well as those currently pursuing strategies that would yield adequately low annual mortality. Under 730-day immune waning, eight states’ current vaccination and treatment coverages would result in less than 10,000 annual deaths if applied nationally, and 11 would result in annual deaths between 10,000 and 50,000. Under 548-day immune waning, only six states have current vaccination and treatment coverages that would result in annual deaths below 10,000 (if those coverages were reached nationally), and three have coverages that would achieve between 10,000 and 50,000 deaths (Fig. 4). Under 365-day immune waning, no state’s coverage would achieve under 10,000 deaths, and one state’s coverage would achieve under 50,000 deaths if applied nationally. Most states are under-vaccinated, with current coverage levels that would be associated > 50,000 annual deaths nationally under 730-day or 548-day waning. Even higher coverage of vaccination and treatment would be needed under 365-day immune waning (Additional file 1: Fig. S11).

### Burden reduction of age-specific vaccination

**Fig. 5:**
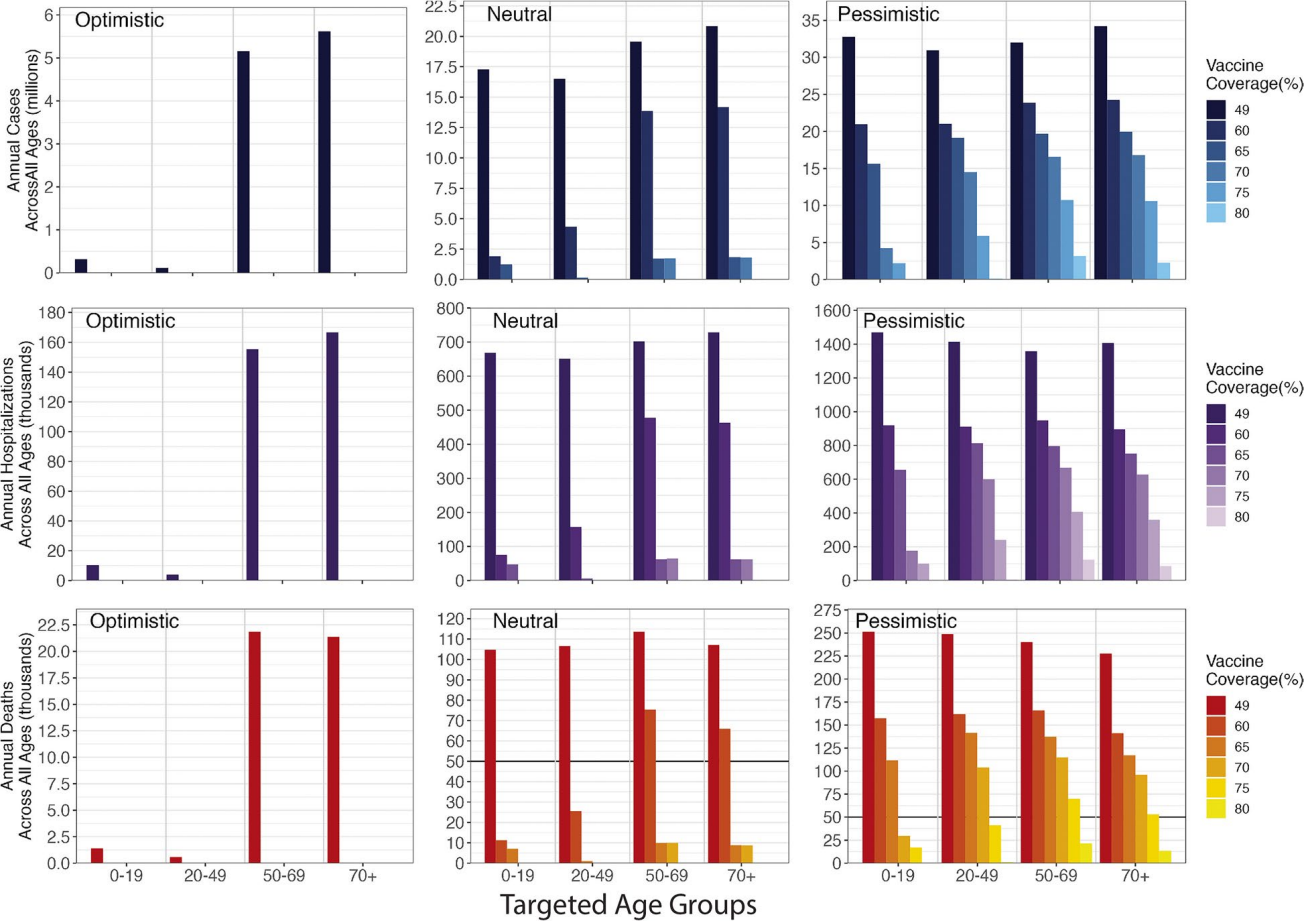
Annual burden across all ages between 2025 and 2033 given age-specific vaccination when immune duration is 548-day. The targeted age group (on *x*-axis) has 25% higher vaccination coverage than the vaccination coverage applied on other age groups. The current vaccination coverage is 49%. The treatment coverage through the entire period is 13.7%

We examined the potential benefits of increasing vaccination coverage in a particular age group by increasing coverage by 25% for each of four age groups: 0–19, 20–49, 50–69, and > 70. Given an immune duration of 548 days, we found that age-specific effects vary across transmission scenarios and the baseline vaccination coverage (Fig. 5). Targeting the age group with the highest population (20–49), the age group with the highest contact rate (0–19) or the age group with the highest death risks (> 70) can all maximize the disease burden reduction under a combination of specific transmission scenario and baseline vaccination coverage. The result suggests a combined consequence from multiple approaches reducing disease burden: either from removing the source of transmission by vaccinating the younger age group or directly protecting the elderly from death by vaccination when the transmission is far from elimination. The findings indicate the necessity for a flexible approach in the management of age-specific vaccination strategies. Evaluation of the current transmission rate and contact rate would be needed to make strategies to maximize the disease burden reduction.

### Potential risks of vaccine or treatment failure

To account for uncertainty in future vaccine and treatment efficacies, we examined additional scenarios to determine how model projections would change if vaccine or treatment efficacies dropped. In all three transmission scenarios, disease burden can still be eliminated after the 2025–2026 season under high coverage of a vaccine with 88% efficacy [53] (this was chosen as the efficacy of primary series of vaccinations against the Delta variant); at least 63–74% coverage would be required under the optimistic scenario (for the three waning rates), 73–98% under the neutral scenario, and 85–98% coverage under the pessimistic scenario for 548-day and 730-day immune duration. For a vaccine with 50% efficacy—to provide an example of an immune-escape scenario like the one seen when the Omicron variant emerged in autumn 2021—universal vaccination would not eliminate mortality burden (Additional file 1: Fig. S12).

Post-treatment viral rebound is a concern for treatment uptake and efficacy, but it is currently rare, seen in < 10% of patients [27–31, 54]. Our projections indicate that if the probability of treatment failure increases to 20%, universal treatment would still be able to reduce mortality by 84% and hospitalizations by 88%, on average, across all three transmission scenarios and three immune durations (Additional file 1: Fig. S13). Furthermore, if the true risk reduction for hospitalization of treatment is 50%, lower than the originally reported 88%, as indicated in recent CDC reports [13], universal treatment is projected to reduce mortality by 82% and hospitalization by 59% on average across all three transmission scenarios and three immune durations. Similarly, using a risk reduction for hospitalization of 30% (as seen in molnupiravir trials [55]), universal treatment is projected to reduce mortality by 77% and hospitalization by 45% on average across all three scenarios and three immune durations (Additional file 1: Fig. S14).

## Discussion

We used a previously validated model to assess future COVID-19 burden under different vaccination and treatment coverages. Our model showed that, if transmission remains unchanged, current vaccine and treatment coverage levels between December 2021 and November 2022 are projected to lead to 81,000–164,600 deaths annually (range: 6700–362,000, across various scenarios of transmission severity and immune durations), a mortality level that exceeds the most severe influenza seasons. As expected [56, 57], simulations of SARS-CoV-2 dynamics through 2033 show that high vaccination coverage and high treatment coverage are both effective approaches for lowering COVID-19 deaths below 50,000 deaths annually—a crucial marker that will allow our public health system to state that control of SARS-CoV-2 is approximately as successful as control of influenza virus.

As we continue the fourth year of the epidemic, COVID-19-associated mortality is still the largest among all infectious pathogens in the US. The next highest annual infectious disease mortality burdens in the US are attributed to influenza virus and pneumonia (between 12,000 and 52,000 deaths annually), respiratory syncytial virus (between 5,000 and 15,000 annual deaths) [58], HIV/AIDS (~ 6,000), and viral hepatitis (~ 5,000) [59]. Clearly, any controllable infectious pathogen that causes > 100,000 annual deaths needs constant prioritization and the full attention of the Centers for Disease Control and Prevention, State Departments of Health, and federal budgeting decisions until annual death numbers can be brought down to levels comparable to other infectious diseases.

Current vaccination coverage in the US remains low for a variety of reasons including lack of access, coordinated disinformation campaigns [60, 61], and vaccine hesitancy [62]. Antiviral therapeutics and monoclonal antibody treatments (Table S4) offer another effective way of reducing COVID-19 hospitalizations and deaths when vaccine hesitancy may be too strong to overcome in the near term. However, as a long-term approach, it is important to remember that treatment offers direct benefits only (to the patient receiving the treatment) while vaccination provides both direct and indirect benefits by lowering the probability of (*i*) one’s own infection and (*ii*) onward infections they would have caused. The increased benefit of vaccination can be seen in Fig. 3, where increasing vaccination alone leads to greater reductions in mortality compared to similar increases in treatment, a finding echoed in other studies [56]. As vaccine coverage increases, the indirect benefits of vaccination do not suffer from diminishing returns; rather, they benefit from accelerating returns [63, 64]. This means that every additional vaccinee lowers total COVID-19 risk more than the previous vaccinee. It is imperative that we remind the nation’s public health leadership of this basic fact of epidemiology as experience from the successful measles, smallpox, and polio vaccination campaigns has faded from memory over the past 75 years. Additionally, although NPIs have proven to be effective [65, 66], they are best used as emergency measures while other interventions are insufficient or not yet available (e.g., during 2020 before COVID vaccines were approved). In a future with endemic COVID-19, vaccination and treatment are the most readily adoptable and sustainable interventions, as they do not require large-scale or long-term behavioral changes such as persistent masking, event cancelations, movement restrictions, and reduced social contacts.

This study contributes to current literature examining the benefits of vaccination coverage to manage COVID-19 [10, 67, 68] while also incorporating treatment coverage [56]. This contrasts with the majority of other treatment-focused studies by examining population benefits rather than individual benefits [69, 70]. Our model provides reliable results through incorporating the key characteristics of long-term COVID transmission in the future, including immunity waning, natural birth, death, aging, and underreporting of the symptomatic cases.

The results of this study can help inform resource prioritization for vaccination and/or treatment. Prioritization can be evaluated at both regional and state levels, as vaccination and treatment coverage vary substantially across and within states (Fig. 4). Identifying states that should be prioritized for increased vaccination and/or treatment access would be an ideal way to initiate a national public health campaign intended to cover the majority of Americans with access to vaccines and COVID-19 treatments. The vertical and horizontal shifts in Figs. S9, S10 and S11 can be used to anticipate changes in total case numbers and absenteeism, strains on healthcare systems, and projected death counts if vaccination and treatment access are inadequate.

Evaluating vaccination and treatment as national coverage percentages does not account for nonuniform access to these interventions and the nonuniform risk of infection and severe outcomes that is already well known for SARS-CoV-2 transmission in the US. Prior to and throughout the COVID-19 epidemic, access to healthcare has been shown to vary substantially among various geographic and demographic groups. It is important to consider implementation strategies that are both effective and equitable [71, 72]. Therefore, we should be careful not to aim absolute or national-level coverage recommendations at an uncertain herd immunity threshold—a mistake made in early 2021 during the initial vaccine rollouts [18]. Recommendations should instead aim for universal access and coverage for both vaccination and treatment through strong and targeted recommendations adapted to every community’s priorities and needs. Passive recommendations for voluntary vaccination have so far proven unable to move annual COVID-19 vaccination past the 50% coverage mark [73].

### Limitations

One notable limitation in our analysis is that we assume that SARS-CoV-2 dynamics in the US will progress in accordance to mixing patterns, reporting rates, and health care access as seen in our RI-based parameterization. The data from RI have the best quality, and previous model fitting was most successful for RI compared to MA, CT, and PA [17, 18]. More than 40 statistical fits were performed during 2020 and 2021 at different stages of data completeness and proved that inference on clinical parameters—progression rates to hospitalization, intensive care unit admission, death—and durations of infection and hospital care is robust across four states [17, 18]. This suggests that the basic clinical progression of a COVID-19 case is similar across states and different public health systems. However, population density, mixing rates, and compliance with NPIs do vary among states and regions in the US. We attempted to address this by linking the situations in the US with RI. We calibrated the national transmission rate based on the national hospitalization data; we scaled national case numbers from RI-case numbers to account for the differences in healthcare situations. But the unaccounted heterogeneity among states and counties will certainly have an effect on the implied herd immunity levels shown in Fig. 3.

Our model considered the susceptible (S) class as not immunologically naïve, instead having some immunity from previous infection or vaccination. The S class represents individuals whose immunity had waned enough to be susceptible to reinfection. This may bias our interpretation of the results as the clinical progression between immune-naïve individuals and individuals with partial immunity is likely different. We attempted to address this by using clinical parameters from studies using populations containing both previously infected and vaccinated individuals (see Table S2). Some studies have shown that vaccine-induced immune protection from severe disease wanes more slowly than immune protection from infection [39], indicating differences in the durations of protection against infection and severe disease. Our model currently only considers protection from infection and therefore is unable to incorporate the likely differences in waning protection from infection compared to waning protection from severe clinical outcomes. Little is known regarding the duration of immunity from severe outcomes, making its incorporation into the model increase uncertainty in burden estimates. Estimates of duration of immunity from infection are varied (Table S1), motivating the consideration of three durations in our study. This is further complicated by the fact that vaccine effectiveness is likely to be heterogeneous across the population and, as in the case of influenza vaccines, varying year to year. To properly address the differences of protection against infection and severe diseases would also require a substantially different model structure from the one we used (Fig S1) and is outside of the scope of our analysis.

We assumed that the duration of immune protection is the same after infection or vaccination; however, it is possible that the waning rates of immunity gained from vaccination and infection differ, though current studies do not appear to have conclusive answers to this question [40]. Incorporating a vaccine that has a different duration of protection compared to natural infection or has a different duration of protection from infection compared to hospitalization or death requires a model structure distinct from the one used in this study as well as further data or assumptions pertaining to those protections. Our results show that average annual mortality differs based on the three durations of immunity presented. Our results may therefore be sensitive to this limitation of model structure and assumptions made. The range of thresholds of vaccination coverage needed to achieve milestones of disease management (such as our thresholds of 10,000 and 50,000 annual deaths) further underlines the important effect of immune waning when forecasting future epidemic waves of SARS-CoV-2.

### Future directions

Increasingly, rebound reports during Paxlovid treatment are being published [27–31, 54, 74]; however, some studies also show rebound occurring during natural infection [74–76]. Even accounting for rebound effects, our model results still indicate that high levels of treatment access will lead to substantial reductions in hospitalization and death. We considered other reductions in the risk of hospitalization after treatment (Additional file 1: Fig. S14), as the relative reduction in hospital admissions will vary by treatment and by the control group against which comparison is done. As more data are collected on treatment efficacy, for currently circulating variants and current levels of hospitalization, it will be imperative to update current projections based on the most commonly available and prescribed drugs. As more patients receive antiviral treatments, another important concern will be the potential for emergence of drug-resistant genotypes. Mutations in SARS-CoV-2 following treatment with nirmatrelvir, a component of Paxlovid, have already been observed in the laboratory [77, 78]. Thus, it is important to continue monitoring outcomes of treatment efficacy studies, and policy should be updated based on any major changes in treatment profile, efficacy, or drug resistance.

The costs of various public health measures to increase vaccination or treatment access are likely to differ. The primary focus of this study was reduction of COVID-19 disease burden and not cost-effectiveness, and the same reduction in mortality achieved through modifying either treatment or vaccination is viewed as equivalent despite having potentially different financial costs. Detailed costing studies will be needed to evaluate the cost-effectiveness of various combinations of vaccine and treatment coverage in reducing morbidity and mortality.

In the first months of the fourth epidemic year (March 2023–July 2023), a total of 18,975 deaths were reported by the CDC [1]. In 2021 and 2022, these months saw low COVID-19 burden compared to other times of year, making this estimate a likely minimum value if incidence trends follow those of the previous years and if an anticipated increase in transmission during winter months is observed. Applying this five-month total to the remainder of the year leads to an estimated 45,540 deaths by the end of February 2024. If the remainder of the year follows these 5 months, then COVID-19 will result in at least as many deaths as the most severe influenza seasons. However, this would align more closely with our scenarios that assume transmission lower than or equal to that in the 2022–2023 season as well as a longer duration of immunity. Observing mortality through February 2024 is crucial to determining which of our scenarios are most likely, informing best practices for future vaccination and treatment strategies.

## Conclusions

A total of 136,000 Americans were reported to have died by the end of the third year of the COVID-19 epidemic, a fact that must be viewed as a public health failure given (*i*) the availability of vaccines in 2022, (*ii*) an update to include the Omicron variant in a bivalent vaccine formulation, and (*iii*) the vaccines’ approval in June 2022 for children under five—the last age group to be vaccinated. Although it is still not clear whether the planning of new and active public health campaigns should aim to get vaccination coverage levels to 80% or 90% or higher, it is clear that the vast majority of US states are under-vaccinated, and it is probable that > 100,000 Americans will die annually from COVID if no major improvements are made in vaccine adoption. The simplest approach to narrowing this gap appears to be a recommendation for universal vaccination and a measurement each year of how effectively this recommendation (and its associated efforts and policies) is working in different states and age groups. Although it is unlikely that changes in vaccination coverage can be achieved quickly, we would strongly urge our national-level public health leadership to begin making plans for outreach and communication around universal COVID-19 vaccine coverage.

### Supplementary Information

**Additional file 1: Text S1.** Estimating current and future vaccine coverages. **Table S1.** Estimated durations of immunity following vaccination or infection from literature. **Table S2.** Updated clinical parameters for the Omicron variant. **Table S3.** COVID-19 vaccine doses administered by age group in the United States between December 1, 2021-November 30, 2022. **Table S4.** Treatment efficacies of COVID-19 therapeutics. **Figure S1.** Model diagram. **Figure S2.** Calibrated output compared to observed data in RI and the US. **Figure S3.** Cumulative doses of COVID-19 vaccines administered in the United States. **Figure S4.** Coverage of Paxlovid in 50 states as of Dec 11, 2022. **Figure S5.** Vaccination coverage in 50 states between Dec 1, 2021, and Nov 30, 2022. **Figure S6.** Weekly percentages of achieved coverage of influenza vaccination by age groups by calendar month. **Figure S7.** Burden reduction slopes following treatment coverage under different vaccination coverages. **Figure S8.** Heatmaps of annual mortality under combinations of vaccine and treatment coverage under each transmission scenario and rate of immune waning. **Figure S9.** Heatmaps of annual hospitalizations under combinations of vaccine and treatment coverage under each transmission scenario and rate of immune waning. **Figure S10.** Heatmaps of annual incident cases under combinations of vaccine and treatment coverage under each transmission scenario and rate of immune waning. **Figure S11.** Combinations of treatment coverage and vaccine coverage that lead to COVID-19 mortality within the range of annual influenza mortality as well as below or over this range. **Figure S12.** Annual burden between 2025 and 2033 given 50% and 88% vaccine effectiveness. **Figure S13.** Annual burden between 2025 and 2033 given 20% probability of treatment failure. **Figure S14.** Annual burden between 2025 and 2033 given 30% and 50% risk reduction to hospitalization after failed treatment.

## Data Availability

Relevant data and code for this study are available at https://github.com/Fuhan-Yang/covid-treatment-psu-cidd.

## Abbreviations

BRFSS: Behavioral risk factor surveillance system
CDC: Center for Disease Control and Prevention
COVID or COVID-19: Coronavirus infectious disease 2019
DOH: Department of Health
HHS: Health and Human Services
NIS-Flu: National immunization survey-flu
NPI: Non-pharmaceutical interventions
RI: Rhode Island
SARS-CoV-2: Severe acute respiratory syndrome coronavirus-2
US: United States
