# Supplementary material for: Benefits of near-universal vaccination and treatment access to manage COVID-19 burden in the United States
Source: BMC Med. 2023 Aug 24;21:321. doi: 10.1186/s12916-023-03025-z (PMC10463609; doi:10.1186/s12916-023-03025-z)
Supplement: Supplementary file 1 — Additional file 1: Text S1. Estimating current and future vaccine coverages. Table S1. Estimated durations of immunity following vaccination or infection from literature. Table S2. Updated clinical parameters for the Omicron variant. Table S3. COVID-19 vaccine doses administered by age group in the United States between December 1, 2021-November 30, 2022. Table S4. Treatment efficacies of COVID-19 therapeutics. Figure S1. Model diagram. Figure S2. Calibrated output compared to observed data in RI and the US. Figure S3. Cumulative doses of COVID-19 vaccines administered in the United States. Figure S4. Coverage of Paxlovid in 50 states as of Dec 11, 2022. Figure S5. Vaccination coverage in 50 states between Dec 1, 2021, and Nov 30, 2022. Figure S6. Weekly percentages of achieved coverage of influenza vaccination by age groups by calendar month. Figure S7. Burden reduction slopes following treatment coverage under different vaccination coverages. Figure S8. Heatmaps of annual mortality under combinations of vaccine and treatment coverage under each transmission scenario and rate of immune waning. Figure S9. Heatmaps of annual hospitalizations under combinations of vaccine and treatment coverage under each transmission scenario and rate of immune waning. Figure S10. Heatmaps of annual incident cases under combinations of vaccine and treatment coverage under each transmission scenario and rate of immune waning. Figure S11. Combinations of treatment coverage and vaccine coverage that lead to COVID-19 mortality within the range of annual influenza mortality as well as below or over this range. Figure S12. Annual burden between 2025 and 2033 given 50% and 88% vaccine effectiveness. Figure S13. Annual burden between 2025 and 2033 given 20% probability of treatment failure. Figure S14. Annual burden between 2025 and 2033 given 30% and 50% risk reduction to hospitalization after failed treatment. [file 12916_2023_3025_MOESM1_ESM.pdf]

Supplemental Materials:

Benefits of near-universal vaccination and treatment access to manage COVID-19 burden in the United States

Fuhan Yang[1], Thu N-A Tran[1], Emily Howerton[1], Maciej F Boni[1]*, Joseph L Servadio[1]*

[1]Department of Biology and Center for Infectious Disease Dynamics, Pennsylvania State University, University Park, PA, 16802, United States

*corresponding authors: Maciej F Boni (mfb9@psu.edu), Joseph L Servadio (jjs7684@psu.edu)

S1 Text. Estimating current and future vaccine coverages

To inform our estimates of annual vaccine coverage, we used data on COVID-19 vaccinations from CDC [22] between December 1, 2021 and November 30, 2022 (Table S3, Fig S3). These data include the total number of doses administered (including doses of two-dose courses, doses of a one-dose courses, or booster doses), total number of first doses administered (including one dose from two-dose courses from Pfizer or Moderna. We assumed that one-dose courses from Johnson & Johnson are included), total number of people completing two-dose courses, total number of additional doses (including an additional dose from two-dose courses, additional dose from J&J, or a non-bivalent booster) administered on people who completed primary series (two-dose courses from Pfizer or Moderna or one-dose series from J&J), total number of second boosters administered, and total number of bivalent boosters administered.

We aimed to estimate the number of individuals who had received any COVID-19 vaccine dose to represent national annual coverage. Because the timing of individual-level booster shots is not provided, it is difficult to know exactly how many individuals in the US received at least one dose of a COVID-19 vaccine during this time period. To account for this, we generated two vaccine coverage estimates based on conservative and optimistic assumptions about which fields represent duplicate doses (i.e., multiple doses were given to the same individual during our one-year period).

1. Conservative: this estimate assumes that all individuals competing their two-course series received the first dose of this series during the time period, and that all second boosters were given to individuals who received a first booster during the time period (i.e., $d_{cons} = d_{admin} - d_{series} - d_{second}$). In a population of nearly 332 million, this yields a conservative coverage estimate of 36%.

2. Optimistic: this estimate assumes that the only duplicate doses were those individuals completing their two-course series (i.e., $d_{opt} = d_{admin} – d_{series}$). This yields an optimistic coverage estimate of 49%.

Future trends of COVID-19 vaccination uptake are assumed to follow influenza vaccination. The average proportion of vaccines administered in each month was calculated, then linearly interpolated to generate weekly estimates of vaccination rates. Because the age groups (6 months – 4 years-old; 5-12 years-old, 13-17 years-old, 18-49 years-old, 50-64 years-old, and 65 years-old and above) in the data are different from the model, the age-specific number of new vaccinees were calculated from the coverage rate and the US census in 2020, and were allocated to the age groups in our model under an assumption of uniformity within each of the above age brackets. The weekly relative coverage rate was calculated by dividing the coverage rate this week by the total coverage achieved in the year. The age-specific annual relative coverage rate is used as the future trend of COVID-19 vaccination coverage.

Table S1. Estimated durations of immunity following vaccination or infection from literature.

ref	Article	Duration	% immune (inf)	% immune (vax)	Location	Time	Notes
[35]	Andrews et al, 2022 (NEJM)	2-4 weeks		82.8	England	Nov 2020 - Jan 2022	ChAdOx1 against Delta
		25+ weeks		43.5			
		20-24 weeks		none			ChAdOx1 against Omicron
		2-4 weeks		65.5			
		15-19 weeks		15.4			
		25+ weeks		8.8			mRNA-1273 against omicron
		2-4 weeks		75.1			
		25+ weeks		14.9			
		2-4 weeks		62.4			BNT162b2 booster after ChAdOx1
		10+ weeks		39.6			
		2-4 weeks		70.1			mRNA-1273 booster after ChAdOx1
		5-9 weeks		60.9			

		5-9 weeks		46.7			ChAdOx1 booster after ChAdOx1
		2-4 weeks		67.2			BNT162b2 booster after BNT162b2
		10+ weeks		45.7			
		2-4 weeks		73.9			mRNA booster after BNT162b2
		5-9 weeks		64.4			
		2-4 weeks		64.9			BNT162b2 booster after mRNA-1273
		2-4 weeks		66.3			mRNA-1273 booster after mRNA-1273
[36]	Ferdinands et al., 2022 (BMJ)	4-6 months		86	United States	Jan 2021 - Jul 2022	VE of 2 doses against ED or urgent care visit against Delta
		6-8 months		79			
		10-12 months		66			
		4-6 month		88			VE of 3 doses against ED or urgent care visit against Delta
		4-6 month		37			VE of 2 doses against ED or urgent care visit against Omicron
		6-8 months		30			
		10-12 months		35			

		12-14 months		16			
		16-18 months		22			
		4-6 months		46			VE of 3 doses against ED or urgent care visit against Omicron
		6-8 months		26			
		8+ months		17			
[37]	Tartof et al., 2021 (Lancet)	5+ months		47	United States	Dec 2020 - Aug 2021	VE of fully vaccinated against infection, 12+
				43			VE of fully vaccinated against infection, 65+
		4+ months		53			VE of fully vaccinated against Delta
				67			VE of fully vaccinated against non-Delta
[38]	De Giorgi et al., 2021 (JID)	11 months	63		United States	Apr 2020 - Feb 2021	Detectable neutralizing titers
[39]		4 months		71.4	International		

	Ssentongo et al., 2022 (BMCID)	5 months		21.8		Dec 2019 - Nov 2021 (published dates)	Systematic Review and meta analysis (18 articles)
[40]	Bobrovitz et al., 2023 (Lancet ID)	3 months	65.2		International	Jan 2020- Jun 2022 (publication date)	Systematic review and meta analysis (11 studies)
		12 months	24.7				
		15 months	15.5				
		3 months	69				Hybrid immunity with primary series vax
		12 months	41.8				Hybrid immunity with primary series vax
		3 months	68.6				Hybrid immunity with booster
		6 months	46.5				Hybrid immunity with booster
[41]	Hansen et al. 2021 (Lancet)	3-6 months	80.5		Denmark	Sep - Dec 2020	Compared rates of Covid among those with and without infection before June 2020

[42]	Nordstrom et al., 2022 (Lancet ID)	3-6 months	0.9696		Sweden	March 2020 - Oct 2021	Infection vs no immunity
		6-9 months	92				
		2+ months		45			one dose hybrid vs natural infection only
		2+ months		56			two dose hybrid vs natural infection only

Table S2: Updated clinical parameters for the Omicron variant. Clinical parameters retained from prior time periods have been previously fitted and published [17, 18]. Citations within cells apply to all parameter values within the cell.

Parameter	Pre-Omicron	Omicron
Incubation period	6 days*[51]	3.42 days*[46]
Infectious period	5 days*	6 days*[47]
Probability of hospitalization by age	0 (0-9 years)†,<br>0.012 (10-19 years)†,<br>0.021 (20-29 years)†,<br>0.030 (30-39 years)†,<br>0.048 (40-49 years)†,<br>0.078 (50-59 years)†,<br>0.147 (60-69 years)†,<br>0.285 (70-79 years)†,<br>0.314 (>=80 years)†	0 (0-9 years)*,<br>0.005 (10-19 years)*,<br>0.021 (20-29 years)*,<br>0.024(30-39 years)*,<br>0.021 (40-49 years)*,<br>0.038 (50-59 years)*,<br>0.056 (60-69 years)*,<br>0.180 (70-79 years)*,<br>0.242 (>= 80 years)* [48]
Mean length of stay in hospital	10.7 days†	13.3 days*[48]
Probability of ICU admission in hospital	0.304 (0-9 years)†,<br>0.293 (10-19 years)†,<br>0.283 (20-29 years)†,<br>0.301 (30-39 years)†,<br>0.463 (40-49 years)†,<br>0.4245 (50-59 years)†,<br>0.46 (60-69 years)†,	0.152 (0-9 years)*,<br>0.1465 (10-19 years)*,<br>0.141 (20-29 years)*,<br>0.151 (30-39 years)*,<br>0.232 (40-49 years)*,<br>0.212 (50-59 years)*,<br>0.23 (60-69 years)*,

	0.484 (70-79 years)†,<br>0.416 (>= 80 years)†	0.242 (70-79 years)*,<br>0.208 (>= 80 years)* [45]
Probability of ventilation in ICU	0.66†	0.238*[48]
Probability of death in non-ICU hospital care	0 (0-69 years)*,<br>0.025 (70-79 years)*,<br>0.050 (>= 80 years)*	0 (0-69 years)*,<br>0.005 (70-79 years)*,<br>0.011 (>= 80 years)* [48]
Probability of death outside of hospital	0 (0-59)*,<br>0.013 (60-69 years)†,<br>0.042 (70-79 years)†,<br>0.227 (>= 80 years)†	0 (0-59 years)*,<br>0.0013 (60-69 years)*,<br>0.0061(70-79 years)*,<br>0.074 (>= 80 years)* [49, 50]
*: Fixed parameters.<br>†: Fitted parameters from previous studies.		

Table S3: COVID-19 vaccine doses administered by age group in the United States between December 1, 2021-November 30, 2022.

	Value
Total doses administered, d_{admin}	193M
Total first doses administered, d_{first}	34M
Total completed two-course series, d_{series}	31M
Total additional doses administered (non-bivalent), $d_{additional}$	73M
Total second boosters administered (non-bivalent), d_{second}	41M
Total bivalent boosters administered, $d_{bivalent}$	40M

Table S4. Treatment efficacies of COVID-19 therapeutics.

Treatment	Efficacy	Reference
Nirmatrelvir/ritonavir (Paxlovid)	87.8 reduction of severity compared to unvaccinated population (prospective study)	[12]
	50% reduction in hospitalization compared to a population mixed with unvaccinated (15%) and vaccinated (85%) individuals (retrospective study)	[13]
Molnupiravir (Lagevrio)	29.9% reduction of hospitalization or death compared to unvaccinated population (prospective study)	[55]
Remdesivir	86.8% reduction of hospitalization or death compared to unvaccinated population (prospective study)	[14]
Bebtelovimab	36-40% reduction of hospitalization compared to a population mixed with unvaccinated and vaccinated individuals (prospective study)	[15]

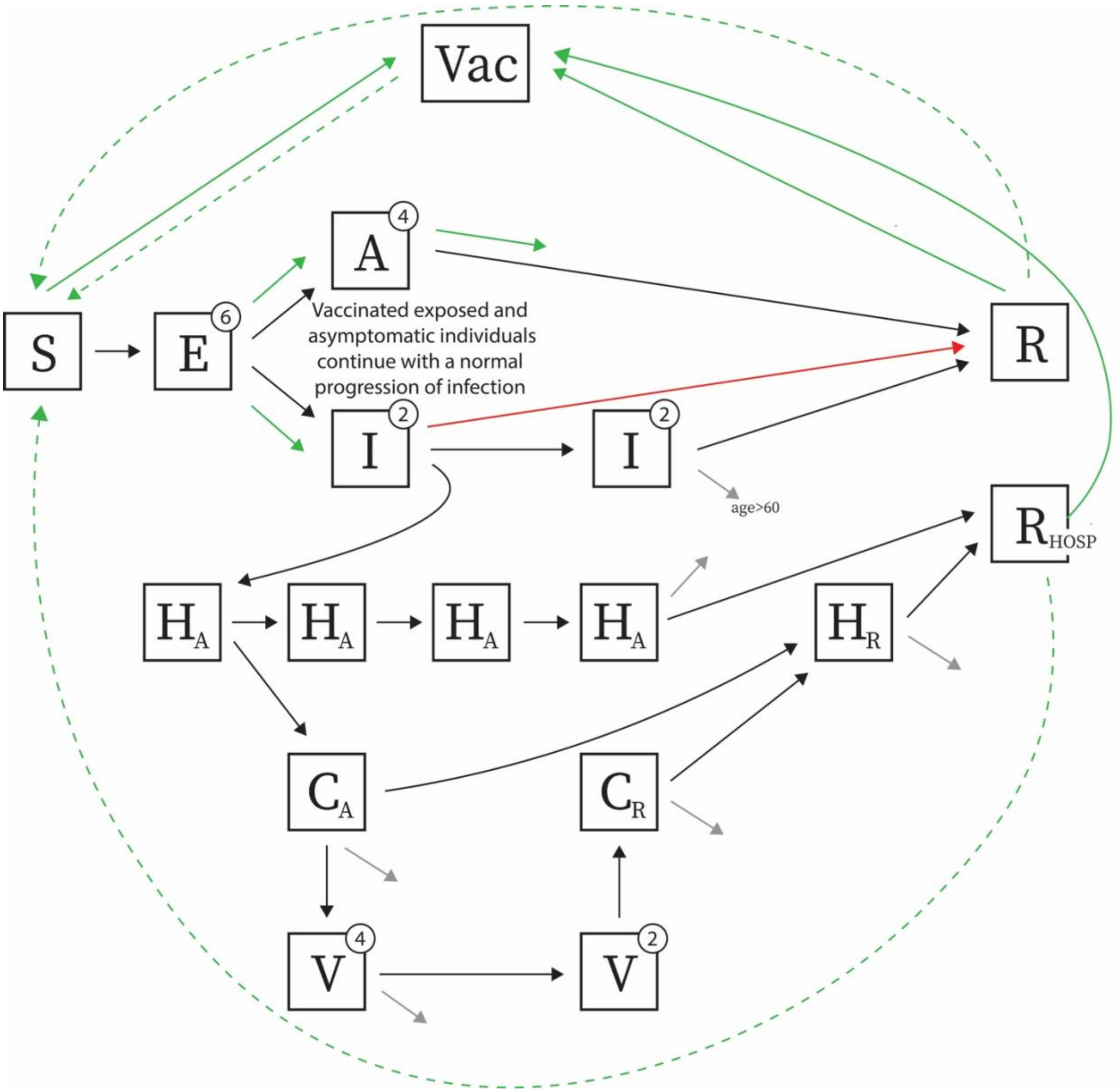

Figure S1. Model diagram. Adapted from a previously published model[17–19] to show movement across compartments. Circled numbers on the upper-right corners refer to the number of repeated compartments (such as having six consecutive Exposed compartments). The grey arrows pointing out of compartments indicate COVID-associated deaths. Changes made include adding a one-stage vaccine compartment (Vac), waning of infection-induced and vaccine-induced immunity, and fast recovery from infection given treatment. The green solid line indicates the vaccine-seeking behavior, where only susceptible and recovered individuals will benefit from vaccination. The green dashed line indicates the waning immunity acquired from infection or vaccination. The red solid line indicates the fast recovery of the infected individuals after successful treatment. Infected individuals with failed treatment continue a normal progress of infection.

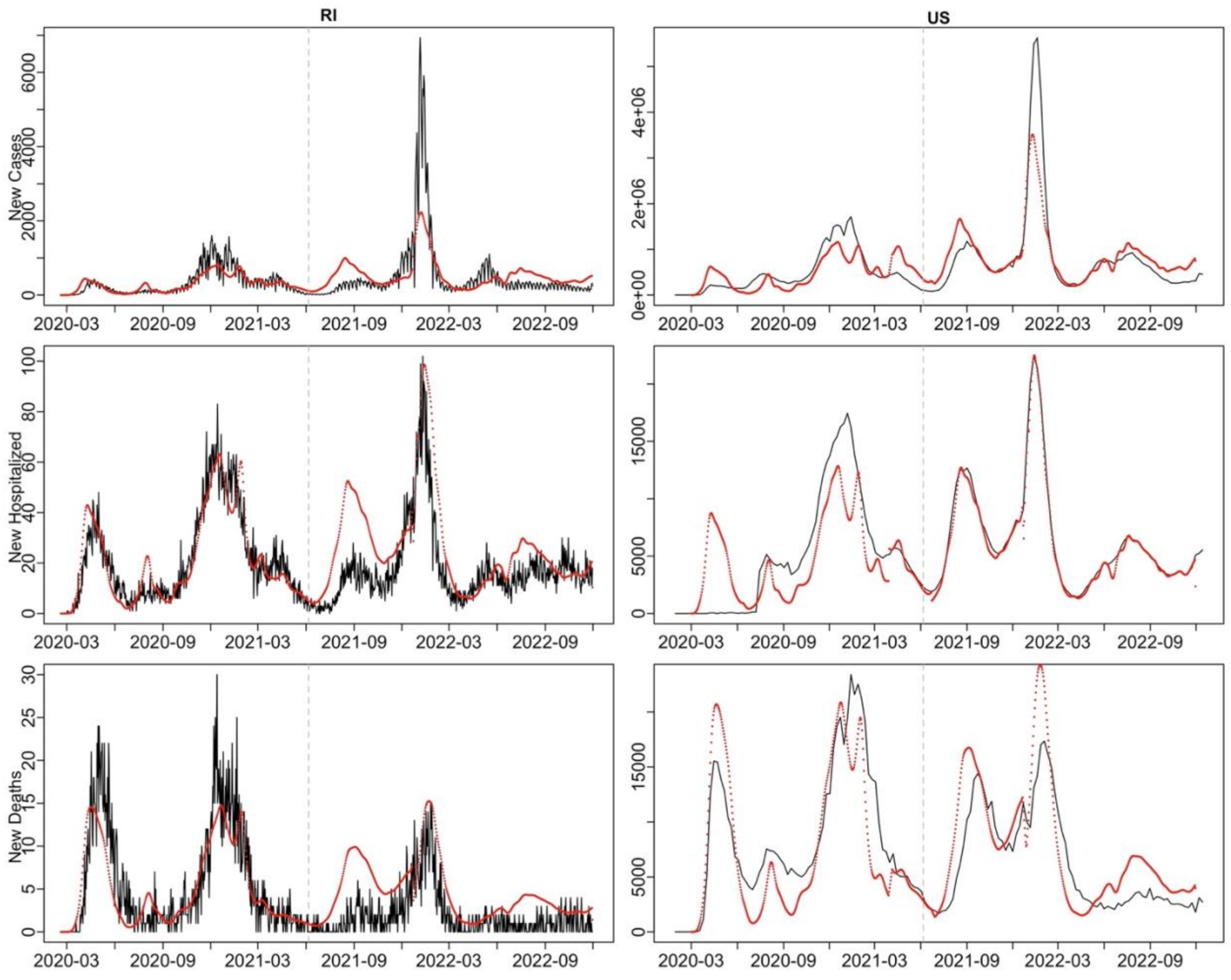

Figure S2. Calibrated output (red points) compared to observed data (black line) in RI (left) and the US (right). After June 6, 2021 (grey vertical dashed line), population mixing rates were calibrated to the hospitalized data in RI. New cases and new deaths in RI were simulated based on calibrated population mixing rates. The new cases, hospitalizations, and deaths in the US were scaled up from RI using the ratio of the total cases in different variant-dominant period between RI and the US. The scaled-up outputs for the US match the trends in the observed data.

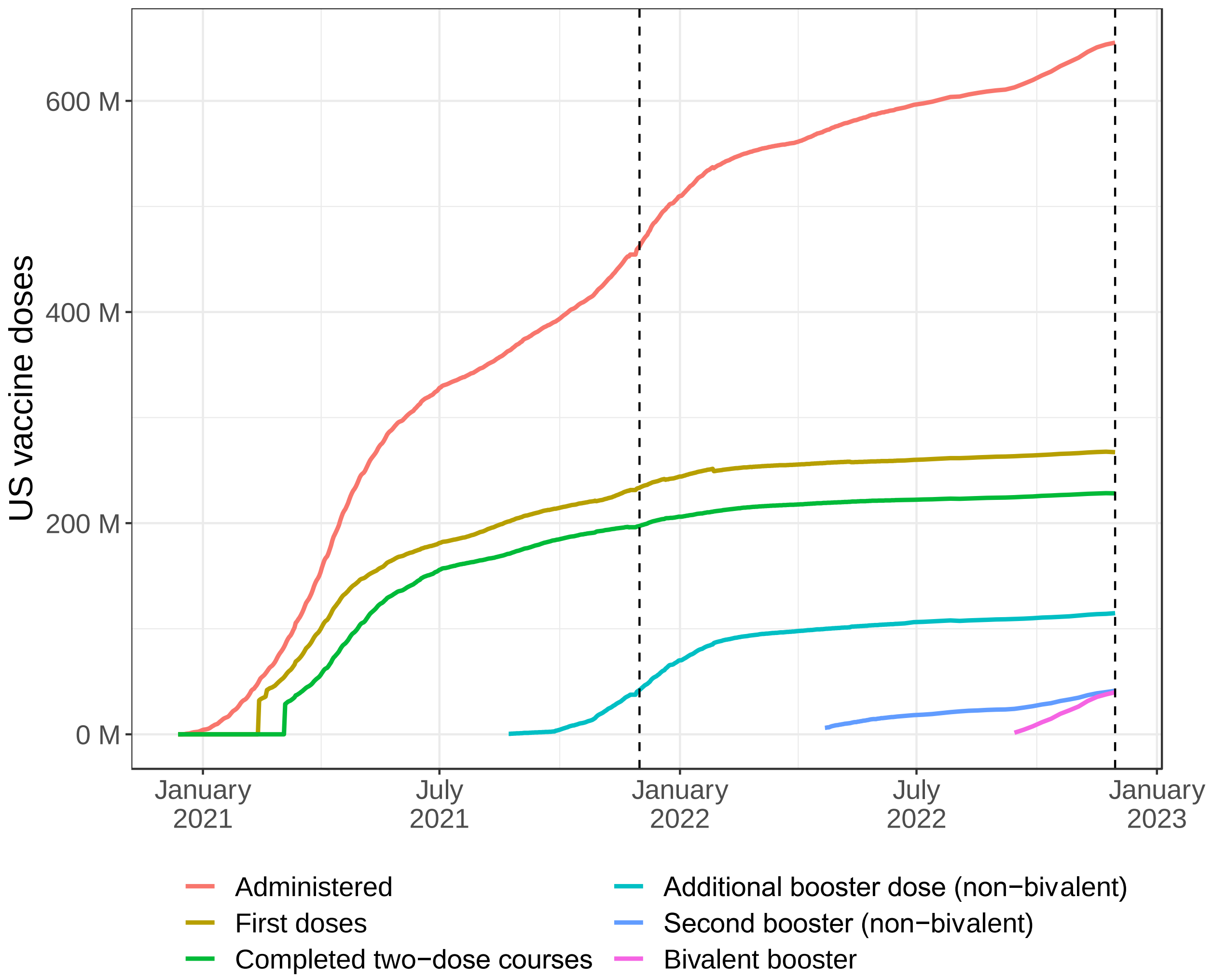

Figure S3. Cumulative doses of COVID-19 vaccines administered in the United States. See detailed description in S1 Text and Table S3. Doses administered between December 1, 2021, and November 30, 2022 (marked with vertical dashed lines) were used to estimate vaccine coverage in the 2022-2023 season.

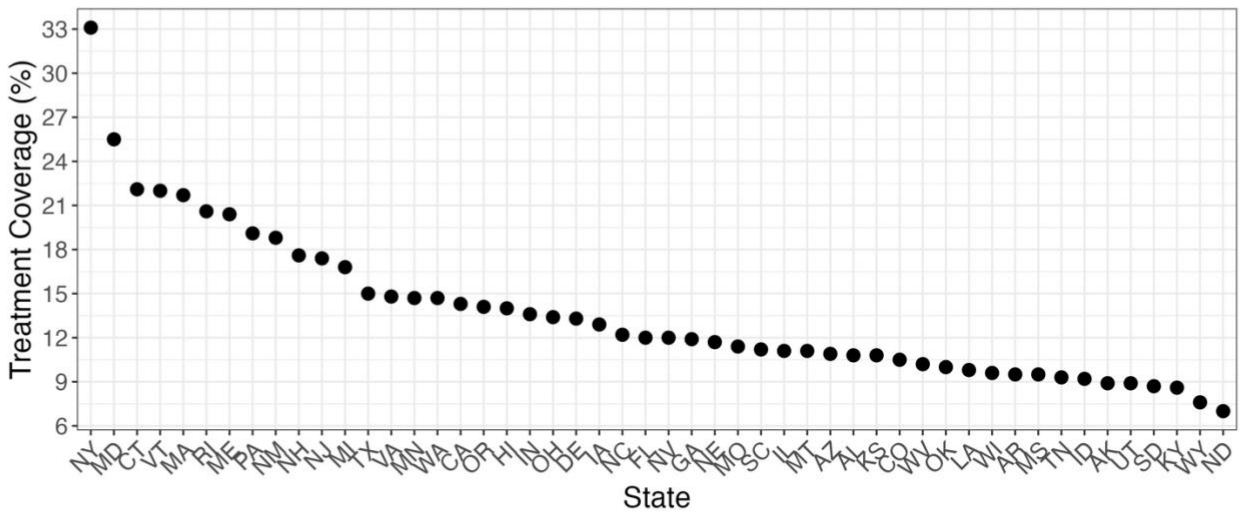

Figure S4. Coverage of Paxlovid in 50 states as of Dec 11, 2022. This is calculated as the cumulative administered courses of Paxlovid on Dec 11, 2022, divided by the number of patients from Jan 1, 2022, to Dec 11, 2022.

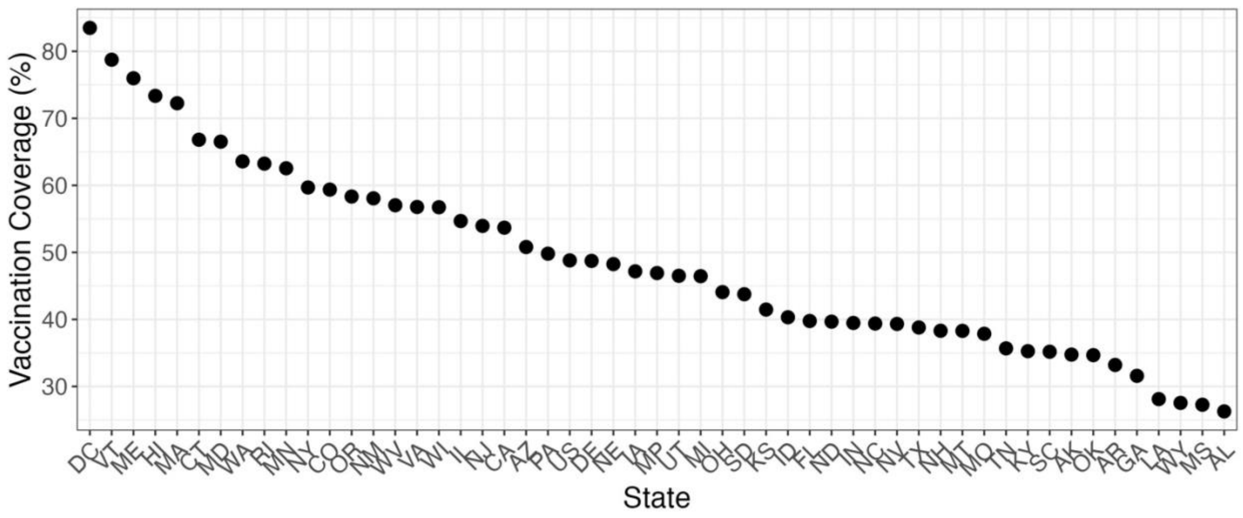

Figure S5. Vaccination coverage in 50 states between Dec 1, 2021, and Nov 30, 2022. Coverage is calculated as the number of administered doses of either a two-course primary series or booster divided by state-wide population (age > 6 months).

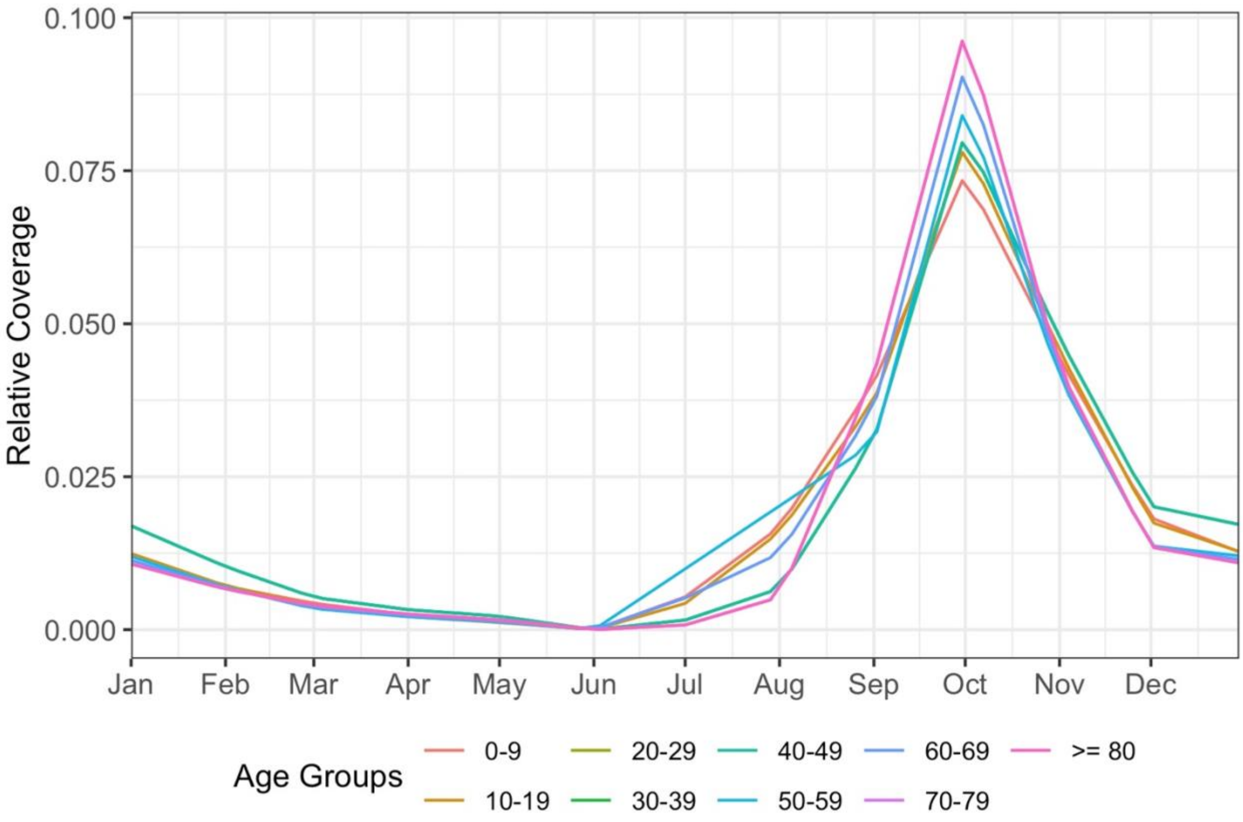

Figure S6. Weekly percentages of achieved coverage of influenza vaccination by age groups by calendar month. The trends observed were applied to COVID-19 vaccination administration in model projections.

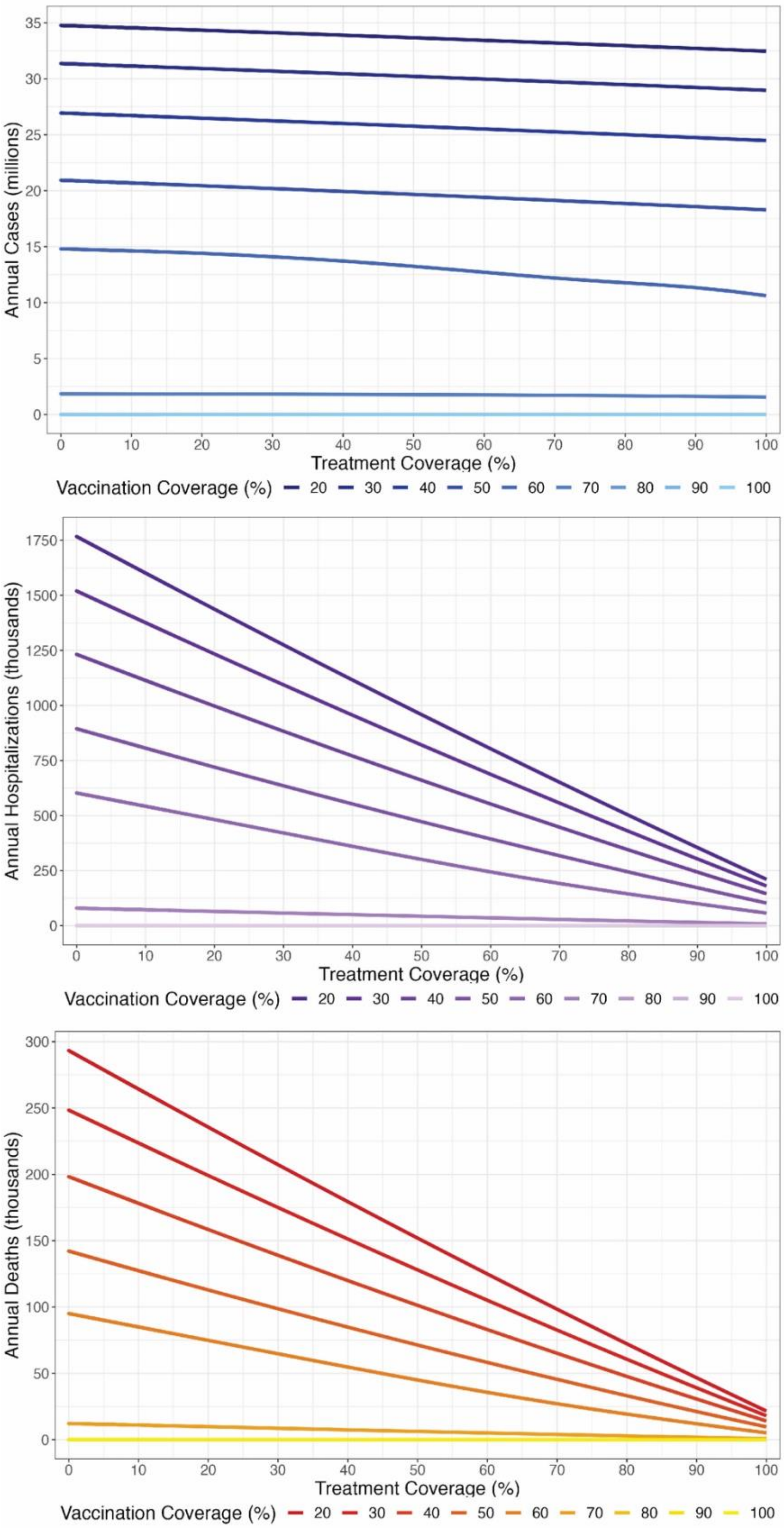

Figure S7. Burden reduction slopes following treatment coverage under different vaccination coverages. Reductions in hospitalizations and deaths are more pronounced under low-vaccination circumstances.

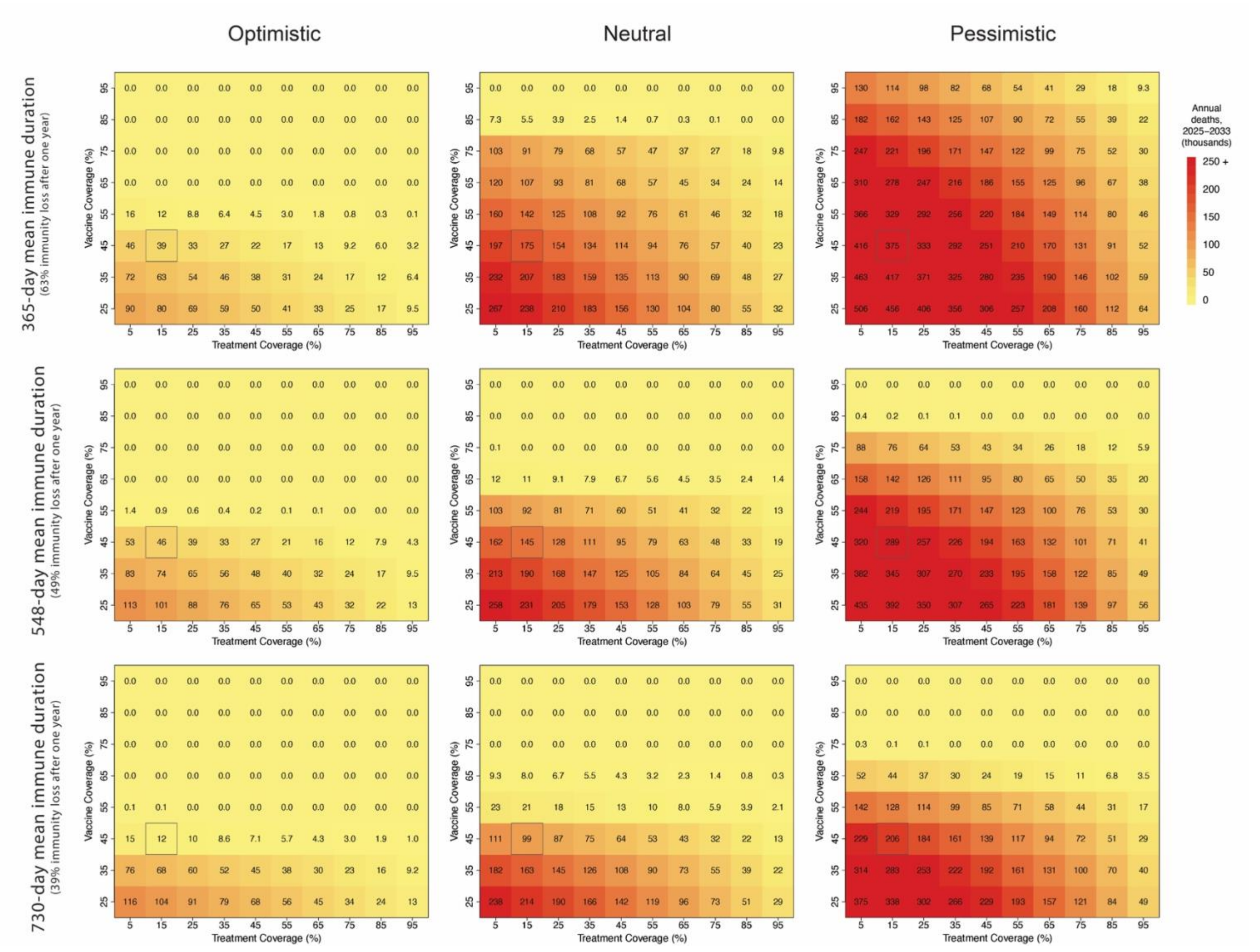

Figure S8. Heatmaps of annual mortality under combinations of vaccine and treatment coverage under each transmission scenario and rate of immune waning.

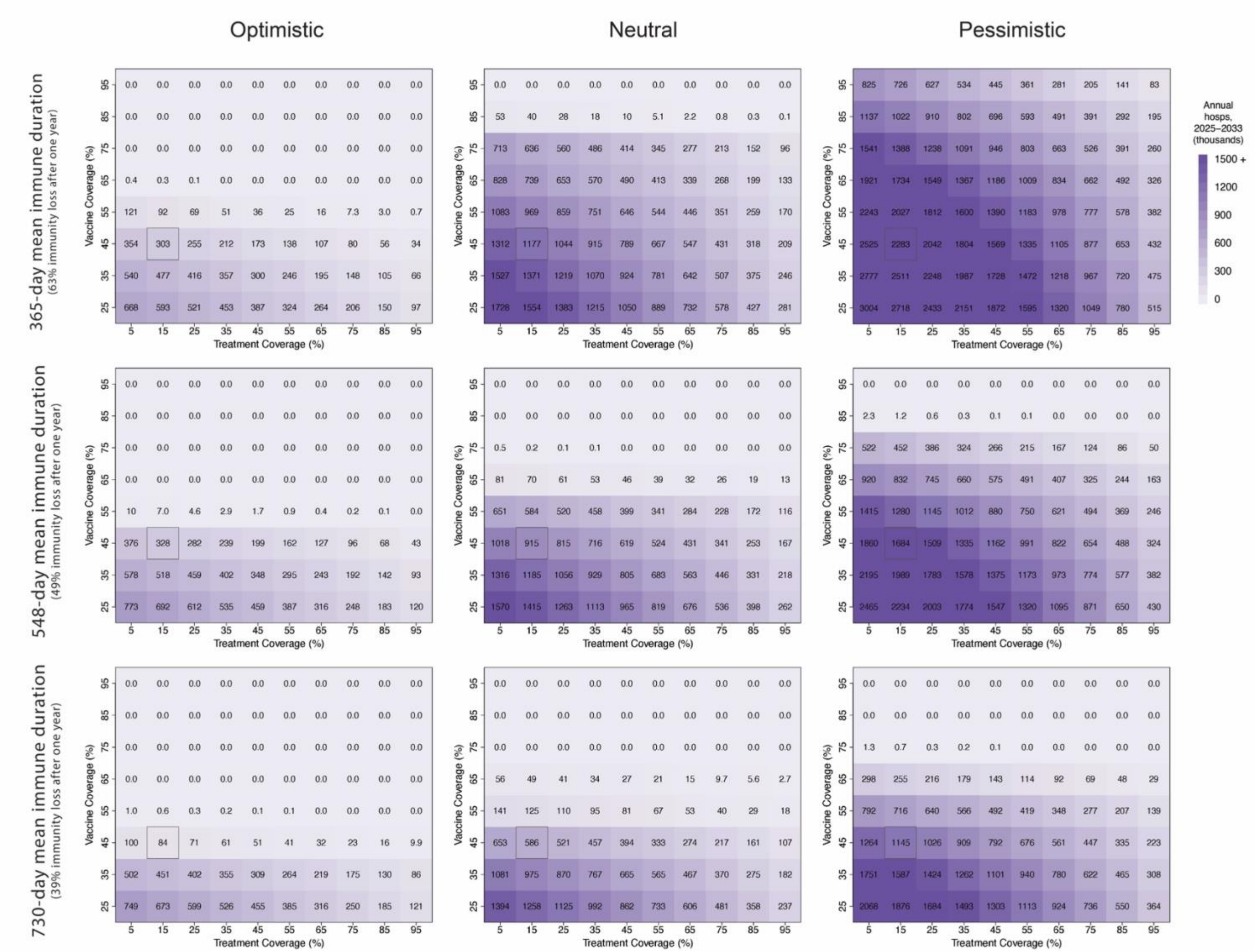

Figure S9. Heatmaps of annual hospitalizations under combinations of vaccine and treatment coverage under each transmission scenario and rate of immune waning.

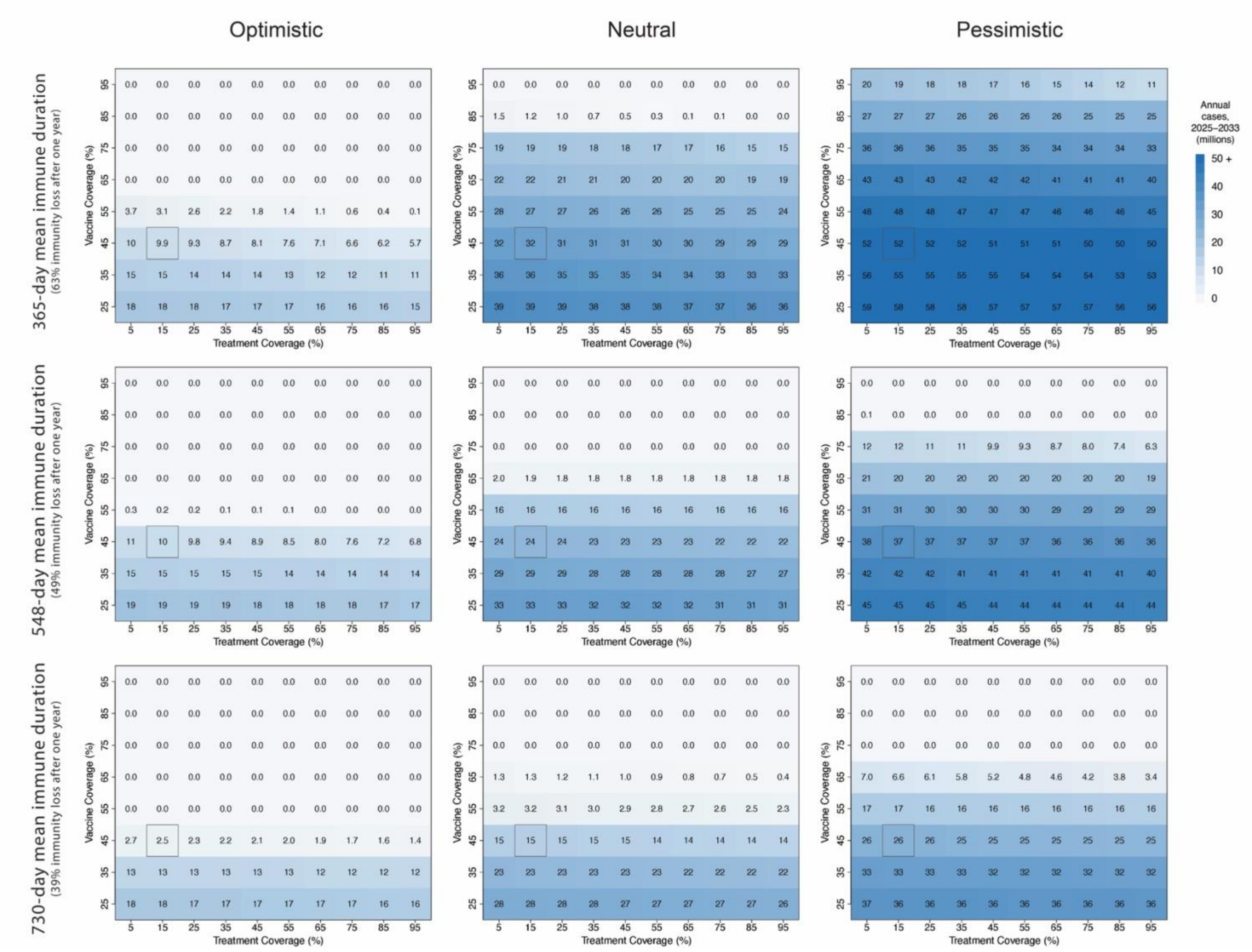

Figure S10. Heatmaps of annual incident cases under combinations of vaccine and treatment coverage under each transmission scenario and rate of immune waning.

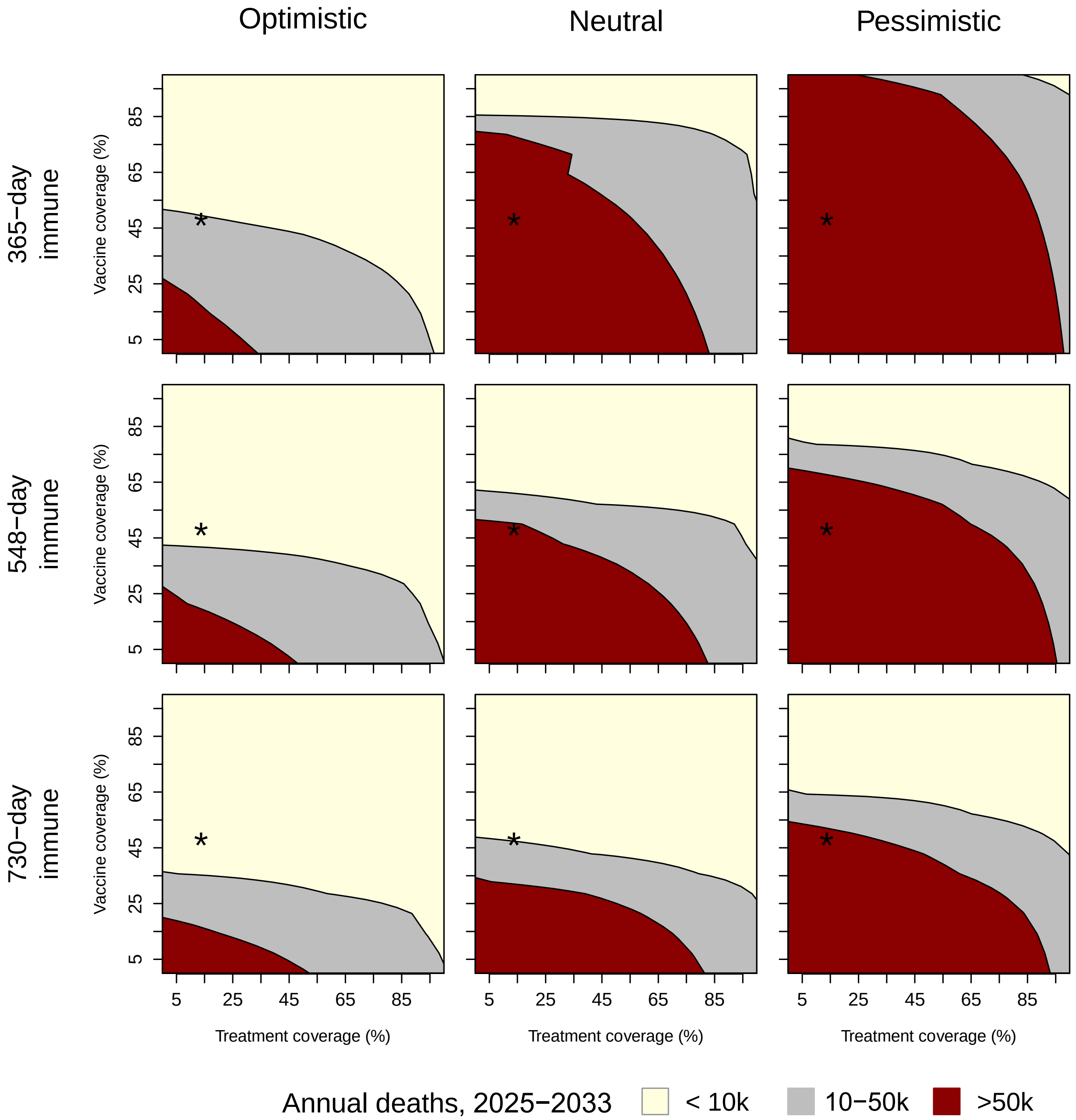

Figure S11. Combinations of treatment coverage (horizontal axis) and vaccine coverage (vertical axis) that lead to COVID-19 mortality within the range of annual influenza mortality (10,000 – 50,000 deaths) as well as below or over this range. The starred point in the plots represents the current treatment and vaccine coverage in the United States.

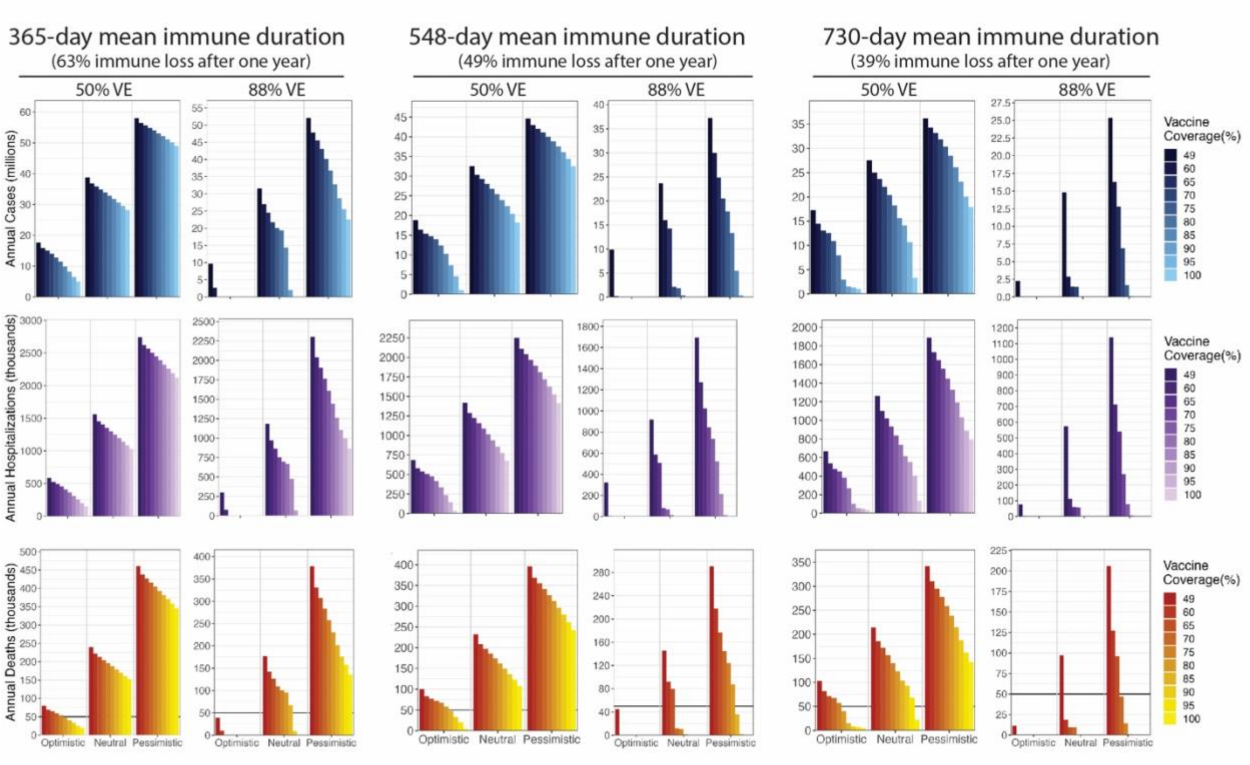

Figure S12. Annual burden between 2025 and 2033 given 50% and 88% vaccine effectiveness. The treatment coverage in the entire period is 13.7%.

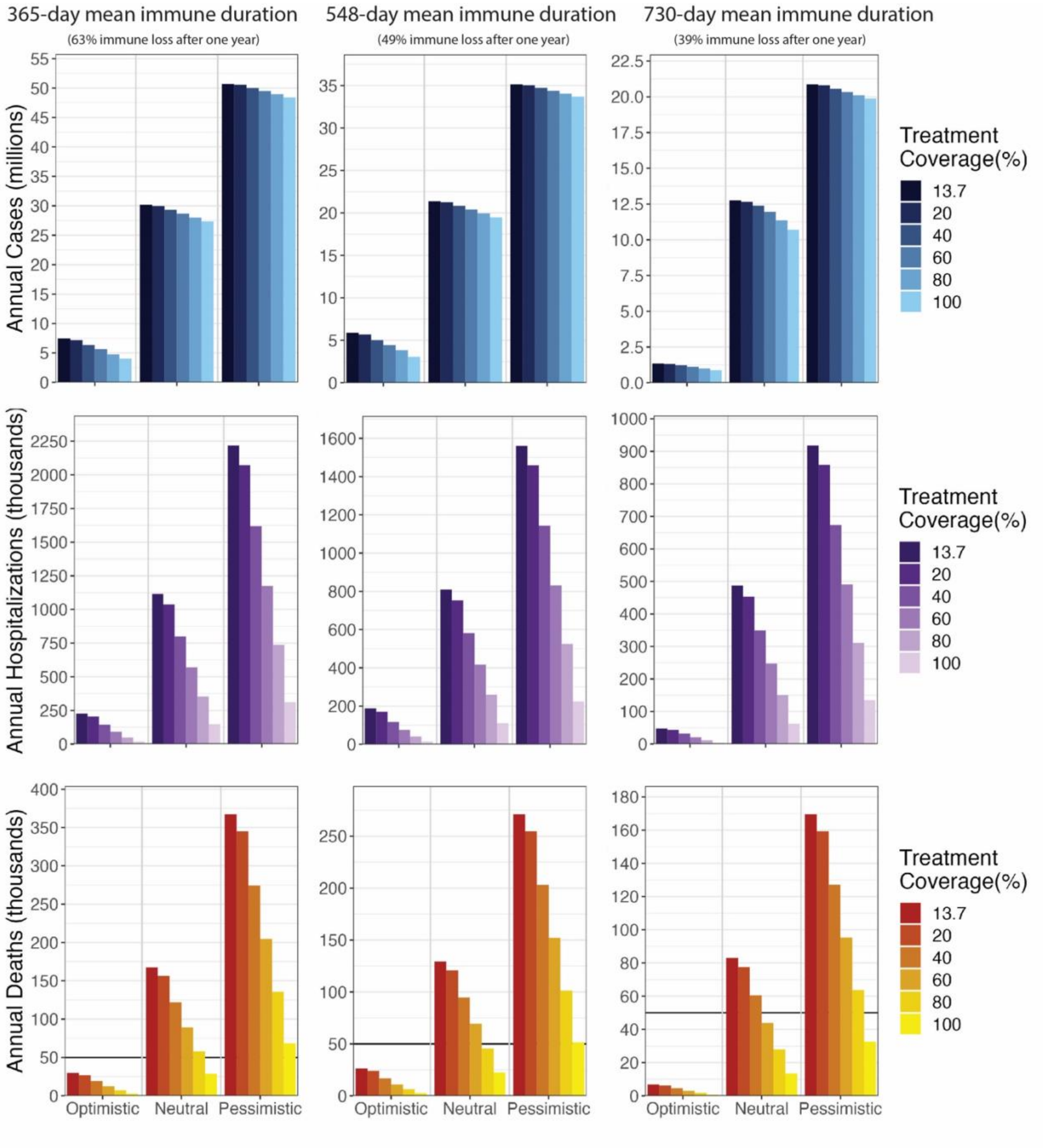

Figure S13. Annual burden between 2025 and 2033 given 20% probability of treatment failure. Vaccination coverage during the entire period is 49%.

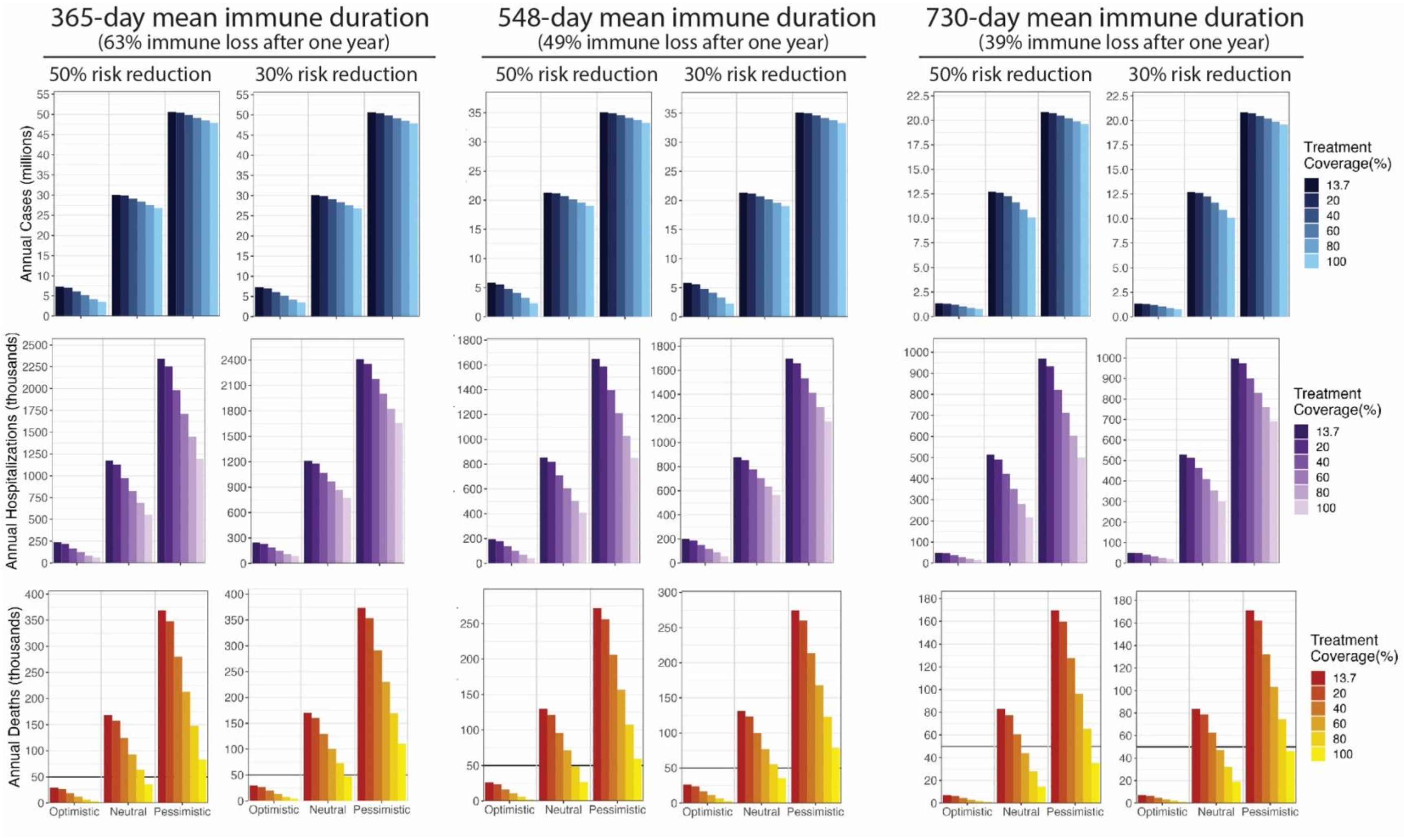

Figure S14. Annual burden between 2025 and 2033 given 30% and 50% risk reduction to hospitalization after failed treatment. The assumed probability of unsuccessful treatment is 0.059. Vaccination coverage during the entire period is 49%.
